# Coral-YOLO: An Intelligent Optical Vision Sensing Framework for High-Fidelity Marine Habitat Monitoring and Forecasting

**DOI:** 10.3390/s25237284

**Published:** 2025-11-29

**Authors:** Jun Tao, Hongjun Tian, Shuai Huang, Yuhan Ye, Yang Xiong, Shijie Huang, Jingbo Qin, Yijie Yin, Jiesen Zhang, Ying Tang, Jiani Wu

**Affiliations:** 1 Engineering College, Shanghai Ocean University, Shanghai 201306, China; 2427219@st.shou.edu.cn (J.T.); 19852242618@163.com (Y.Y.); 13377022951@163.com (Y.X.); 17349768846@163.com (S.H.); bo333644@163.com (J.Q.); silence18177@163.com (Y.Y.); 2 College of Energy and Mechanical Engineering, Shanghai Electric Power University, Shanghai 201306, China; 3355466192@mail.shiep.edu.cn; 3 College of Marine Science and Ecological Environment, Shanghai Ocean University, Shanghai 201306, China; jayson126@126.com (J.Z.); ty1462330430@gmail.com (Y.T.); 17717284858@163.com (J.W.)

**Keywords:** object detection, coral reef monitoring, deep learning, underwater vision, spatio-temporal forecasting, YOLO, attention mechanisms, intelligent monitoring, stochastic learning

## Abstract

Coral reefs are facing a catastrophic decline due to climate-induced bleaching, threatening critical marine biodiversity. Automated, large-scale monitoring is essential; however, modern object detectors are hindered by two fundamental limitations in complex underwater scenes: a spatial reasoning deficit in their decoupled heads, which inhibits robust multi-scale feature integration, and a feature robustness deficit, which renders deterministic networks vulnerable to stochastic visual variations. To address these limitations, we propose Coral-YOLO, a novel framework for detection and forecasting. We introduce the Holistic Attention Block Head (HAB-Head), which enables deep cross-scale reasoning through explicit feature interaction, and MCAttention, a randomized training mechanism that enables the network to learn scale-invariant features with inherent robustness. Evaluated on our newly curated, multi-year CR-Mix dataset, Coral-YOLO achieves a state-of-the-art 50.3% AP (average precision at IoU threshold 0.5:0.95, following COCO metrics), representing a +1.8 percentage point improvement over the YOLOv12-m baseline, with particularly pronounced gains on small objects (+2.6 percentage points in $AP_{S}$). Crucially, its integrated temporal forecasting module achieves 82.7% accuracy in predicting future coral health, substantially outperforming conventional methods. Coral-YOLO sets a new performance benchmark and enables proactive reef conservation. It provides a powerful tool to identify at-risk corals long before severe bleaching becomes visually apparent.

## 1. Introduction

Coral reefs, often referred to as the ‘rainforests of the sea’, constitute foundational components of marine biodiversity and play a pivotal role in global human welfare. Occupying less than 0.2% of the ocean floor, they astonishingly support at least 25% of all marine species and provide an estimated USD 9.9 trillion annually in economic goods and ecosystem services, sustaining hundreds of millions of people worldwide [1,2]. However, these ecosystems are experiencing accelerated degradation. Recent data indicate a substantial decline in live coral cover, with over 80% of the world’s corals having experienced severe bleaching events [3]. This phenomenon, primarily driven by climate change-induced temperature increases and exacerbated by ocean acidification and pollution, is causing rapid degradation of reef structures and significant loss of biodiversity [4]. The urgent need for effective conservation strategies is therefore predicated on our ability to monitor reef health with high spatial and temporal resolution.

Although remote sensing enables large-scale spatial coverage, its applicability is largely confined to shallow waters. This applicability is contingent upon ideal environmental conditions, making in situ visual surveys the de facto standard for high-fidelity reef assessment [5]. The integration of computer vision into these surveys, particularly through deep learning-based object detection, has shown immense promise for automation and scalability [6,7]. However, the unique challenges of the underwater domain—characterized by poor contrast, high noise, and the complex, irregular morphology of coral colonies—reveal a deeper, more fundamental limitation in the design philosophy of modern detectors. These systems excel at feature extraction, employing sophisticated backbones to learn rich hierarchical representations. However, this sophistication is often squandered at the final, critical stage of prediction. We identify a significant architectural gap: the prevailing ‘decoupled head’ design processes multi-scale features from the feature pyramid through largely isolated computational streams [8,9]. This design prevents holistic, cross-scale reasoning, making it difficult, for example, to leverage the contextual information of a large coral colony to correctly identify a tiny, ambiguous bleached patch on one of its branches. Furthermore, these deterministic models are ill-equipped to handle the inherent stochasticity of natural processes, as they cannot learn robust, scale-agnostic feature representations from dynamic visual data.

To address these limitations, this paper proposes a dual-principle detection framework termed Coral-YOLO. We hypothesize that improved performance in complex natural scenes requires two architectural properties: explicit cross-scale reasoning within the detection head and stochastic feature learning throughout the network. We instantiate these principles through targeted architectural modifications to standard YOLO-style detectors. We instantiate these principles through two core architectural innovations. First, we introduce the Holistic Attention Block Head (HAB-Head), a new detection head architecture centered on our original HAB module, which enforces explicit, deep interaction between parallel feature pyramid levels to achieve globally informed, context-aware predictions. Second, we propose Monte Carlo Attention (MCAttention), a novel mechanism that introduces a randomized pooling strategy during training to learn highly robust, scale-invariant features. These core innovations are supported by our unique Stochastic Fourier Dynamic Convolution (SFD-Conv) for adaptive downsampling and a dynamic SD-Loss for scale-aware regression. To provide a clear summary of our contributions, the table below delineates the key architectural and methodological innovations of Coral-YOLO. It contrasts each proposed module with its corresponding component in the YOLOv12-m baseline, clarifying which aspects of our framework are entirely novel additions and which are principled enhancements over existing mechanisms. This provides a concise overview of how Coral-YOLO is architecturally distinct from standard real-time object detectors.

We validate our framework on a scientifically critical and technically challenging task of multi-year coral bleaching monitoring. Our framework is trained and evaluated on a large-scale, time-series coral dataset, meticulously annotated with four fine-grained health states: (1) Healthy, (2) Sub-healthy (representing a state of partial bleaching), (3) Bleached, and (4) Dead. This enables not only high-fidelity detection but also the forecasting of subtle transitions in coral health. Our contributions are fourfold:

1. We identify and analyze a fundamental design limitation in modern object detectors: the lack of explicit cross-scale reasoning at the prediction stage, where conventional decoupled heads process multi-scale features through isolated computational streams.
2. We propose two core, original architectural solutions: the HAB-Head for holistic spatial reasoning and MCAttention for robust, stochastic feature learning.
3. We integrate these into a comprehensive, high-performance detection framework, Coral-YOLO, which our novel SFD-Conv and SD-Loss further enhance.
4. We provide compelling empirical evidence of our framework’s superiority through extensive experiments on a challenging, real-world time-series coral dataset, not only achieving state-of-the-art detection performance but also establishing the first robust framework for temporal bleaching prediction.

The remainder of this paper is organized as follows. Section 2 reviews related work in object detection and ecological monitoring. Section 3 details the proposed Coral-YOLO framework and its core components. Section 4 presents our comprehensive experimental setup, results, and ablation studies. Finally, Section 5 discusses the broader implications of our findings and concludes the paper.

## 2. Related Work

Our work is situated at the intersection of computer vision for ecological monitoring, the architectural evolution of object detectors, and advanced attention mechanisms.

### 2.1. Computer Vision in Marine Ecology

The application of computer vision to automate the monitoring of marine ecosystems, particularly coral reefs, is a rapidly growing field driven by urgent conservation needs [10]. Initial efforts focused on creating benchmarks and applying established models to tasks such as species classification and segmentation [11,12]. Pioneering systems like CoralNet have successfully automated patch-level classification on a large scale, resulting in over 65 million annotations to date [13]. While patch-based classification effectively estimates overall coral coverage, it struggles to accurately delineate the complex boundaries of individual coral colonies. This precision is essential for detailed morphological analysis and object-level tracking. As a result, the field is increasingly focusing on more advanced, end-to-end segmentation models such as CNet and CoralSCOP [14,15]. However, while these models improve accuracy, they often compromise real-time performance, which is crucial for timely ecological assessments and rapid conservation responses [16]. This persistent precision-speed dilemma highlights a critical gap for a framework that is both scientifically rigorous and efficient enough for large-scale, dynamic monitoring. More fundamentally, these works have primarily focused on improving backbone networks, leaving the crucial role of the object detection head in handling extreme scale variation largely underexplored.

### 2.2. The Evolution of Real-Time Object Detection

The YOLO (You Only Look Once) series has defined the state-of-the-art in real-time object detection, consistently advancing the speed-accuracy frontier through relentless architectural innovation [17,18]. The rapid evolution in 2024–2025 has introduced sophisticated mechanisms to address various information bottlenecks: YOLOv9’s Programmable Gradient Information (PGI) mitigates information loss in deep networks [19], YOLOv10 achieves an elegant NMS-free pipeline through dual label assignment [20], YOLOv11 enhances feature extraction with improved C3k2 blocks [21], and YOLOv12 introduces attention-centric architectures [22]. The latest YOLOv13, released in mid-2025, continues this trajectory with further refinements in multi-scale feature fusion [23].

Despite this rapid evolution, a core architectural principle remains largely unchallenged: the use of a Feature Pyramid Network (FPN) [24] to generate multi-scale features, which are then processed by a decoupled head for independent classification and regression [8]. While innovations like PGI, NMS-free designs, and attention mechanisms optimize information flow and post-processing, they do not fundamentally address the spatial reasoning deficit inherent in the decoupled head’s architecture. The decoupled head structure, which processes features from different pyramid levels in parallel but isolated streams, creates an information bottleneck at the prediction stage.

This design structurally prevents explicit cross-scale reasoning—a limitation particularly detrimental in complex natural scenes, such as coral reefs, where objects exhibit vast scale variations and intricate morphologies. The primary reason this issue has not been a central focus in many YOLO studies is that research has, justifiably, prioritized other critical areas: enhancing the feature extraction power of the backbone, optimizing the multi-scale fusion in the neck, and improving end-to-end efficiency. Consequently, while substantial research has optimized YOLO’s backbone and neck structures, the detection head itself—even in the latest YOLOv13—has remained relatively untapped for the fundamental redesign required to resolve this cross-scale reasoning deficit. Our work is, to our knowledge, the first to identify and explicitly address this specific bottleneck in YOLO-style architectures by proposing a new head design that moves beyond the decoupled paradigm.

### 2.3. Attention and Dynamic Mechanisms

To enhance the feature representation capabilities of standard convolutional networks, attention mechanisms and dynamic networks have become indispensable tools for improving feature representation. Seminal works, such as SE-Net, introduced channel attention to recalibrate feature responses, while subsequent efforts combined it with spatial attention for further refinement [25,26,27]. Concurrently, dynamic networks, such as DyConv, proposed generating convolutional kernels conditioned on the input, enabling the model to adapt to varying object characteristics [28,29]. While effective, these mechanisms are typically designed as general-purpose plug-in modules operating under a deterministic assumption. Our work builds upon these foundational ideas but diverges in two critical ways. First, rather than incrementally adding attention modules to an existing head, our HAB-Head fundamentally redesigns the head architecture itself to enforce a structured, holistic, and multi-scale reasoning process. Second, inspired by the stochastic nature of real-world data, our MCAttention module moves beyond deterministic recalibration, introducing a randomized learning strategy to enhance the model’s feature robustness against scale variations explicitly. In concert, our contributions aim to resolve the architectural limitations identified in current detectors, offering a more principled and robust solution for challenging detection tasks.

Our review of the literature, summarized in Table 1, reveals two fundamental, unaddressed challenges in modern object detectors when applied to complex underwater scenes.

First, a spatial reasoning deficit exists in the prevalent decoupled head architecture. While methods like FPN and advanced backbones improve feature extraction, the final prediction stage still processes multi-scale features in isolated streams. This design structurally inhibits the cross-scale reasoning essential for contextual understanding, particularly for small or ambiguous objects found in coral reefs.

Second, a feature robustness deficit arises from the deterministic nature of these models. Standard networks and attention mechanisms rely on a fixed context-to-weight mapping optimized for training data statistics. This makes them inherently vulnerable to the stochastic visual variations (e.g., unpredictable lighting, water turbidity, and scale) that are characteristic of real-world underwater imagery.

This analysis highlights a critical research gap for a framework that is both context-aware and robust to stochasticity. Therefore, this paper proposes Coral-YOLO to address these two deficits directly. Our work introduces the HAB-Head to solve the spatial reasoning deficit by enforcing holistic feature interaction at the prediction stage, and the MCAttention module to tackle the feature robustness deficit by learning invariance to contextual randomness. By doing so, we aim to establish a new state-of-the-art in automated coral reef monitoring.

## 3. The Proposed Coral-YOLO Framework

Modern object detectors face two architectural bottlenecks when applied to complex natural scenes: a spatial reasoning deficit and a feature robustness deficit. The prevalent decoupled head paradigm, by processing multi-scale features in isolated streams, structurally inhibits the cross-scale reasoning essential for contextual understanding. Concurrently, the deterministic nature of standard networks renders them inherently vulnerable to the stochastic visual variations—such as lighting, clarity, and scale—found in real-world environments, including coral reefs.

To systematically dismantle these bottlenecks, we construct our framework, Coral-YOLO, upon two foundational design principles. First, the Principle of Holistic Prediction, which mandates that the final prediction at any scale must be explicitly conditioned on the full context of all available scales. Second, the Principle of Stochastic Feature Learning, which posits that actual robustness is achieved by training the network to be invariant to feature-level randomness.

The following sections detail the architectural materialization of these principles. We first present the overall framework, then deconstruct our core innovations—the HAB-Head and MCAttention—demonstrating how they directly derive from these principles to achieve a new level of detection performance.

### 3.1. Overall Architecture

**Figure 1:**
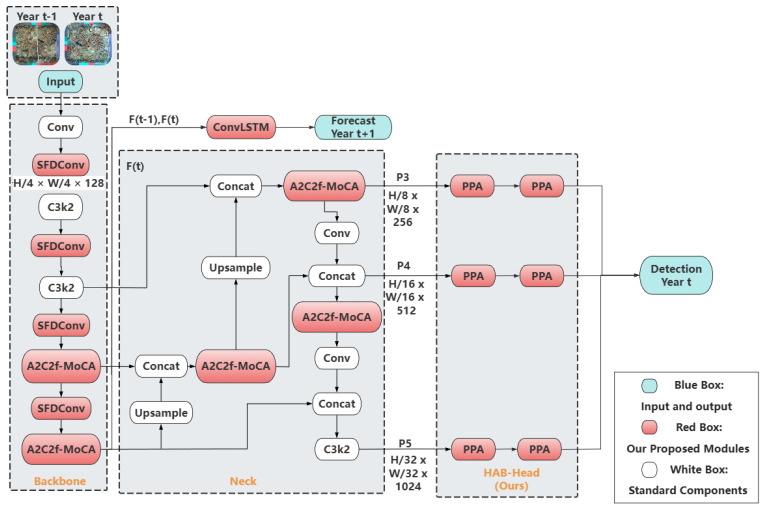
The overall architecture of the proposed Coral-YOLO framework. Our innovations (highlighted in red) are integrated to process a temporal image sequence for both detection and forecasting tasks.

The Coral-YOLO framework, illustrated in Figure 1, implements our design principles through four targeted architectural modifications to the YOLOv12-m baseline. These modifications collectively address the identified spatial reasoning and feature robustness deficits while maintaining compatibility with standard YOLO training pipelines.

The architecture is built upon three primary pillars of innovation for its detection pipeline, supplemented by a dedicated forecasting branch:

1. A Redesigned Head for Holistic Prediction: To address the critical spatial reasoning deficit of conventional detectors, we completely replace the standard decoupled head. Our new Holistic Attention Block Head (HAB-Head) is explicitly engineered to realize our Principle of Holistic Prediction. Unlike a standard head that processes multi-scale features in isolated streams, the HAB-Head employs a deep, multi-path architecture that forces explicit interaction between features from different receptive fields within each prediction level. This deep contextual reasoning, detailed in Section 3.2, enables the model to leverage the full context of the scene (e.g., using a large colony to identify a small patch on its branch), dramatically improving performance on challenging, small, or partially occluded objects.
2. Robust Feature Learning Blocks: To combat the feature robustness deficit caused by stochastic underwater visual variations, we introduce our Monte Carlo Attention (MCAttention) modules. These modules are seamlessly integrated into the network’s deep backbone and neck using a novel block structure, termed A2C2f_MoCA. Operating on our Principle of Stochastic Feature Learning, MCAttention deliberately introduces randomness into the attention mechanism’s context generation process only during training. As detailed in Section 3.3, this forces the model to learn intrinsic, invariant feature relationships that are robust to statistical shifts in the input data, resulting in a model that generalizes better to unseen environmental conditions.
3. An Adaptive Backbone Foundation: To ensure that the features supplied to the neck and head are of the highest possible quality, we enhance the backbone’s foundational layers. We replace the standard, static strided convolutions used for downsampling with our novel Stochastic Fourier Dynamic Convolution (SFD-Conv). Standard convolutions apply a fixed, learned kernel to all inputs, whereas SFD-Conv dynamically generates a unique, sample-specific downsampling kernel for each input image. As explained in Section 3.4.1, this mechanism allows the backbone to adapt its feature aggregation strategy based on the specific characteristics of each scene, creating a more flexible and data-driven foundation for the entire network.

To provide a clear map of the data flow, let us walk through the process as illustrated in Figure 1. The framework operates on a temporal sequence of images and processes them through two parallel streams for detection and forecasting:

Temporal Input and Shared Backbone: The model takes a sequence of two images, from Year t − 1 and Year t, as input. Both images are first processed by a shared backbone, which is enhanced with our novel Stochastic Fourier Dynamic Convolution (SFD-Conv) for adaptive downsampling. This backbone generates high-level feature representations, F(t − 1) and F(t).

Parallel Processing Streams: After the backbone, the feature representations are channeled into two distinct, parallel pipelines:

(a) The Detection Stream (for Year t): The feature map from the current year, F(t), flows into a PANet-style neck. Our Monte Carlo Attention (MCAttention) modules are strategically integrated into the deeper layers of both the backbone and neck to learn robust, scale-invariant features. The resulting multi-scale feature pyramids are then processed by our novel Holistic Attention Block Head (HAB-Head), which replaces the standard decoupled head. The HAB-Head performs deep, cross-scale reasoning to generate the final, highly accurate bounding box detections for the current year, t.
(b) The Forecasting Stream (for Year t + 1): In parallel, the sequence of high-level features, [F(t − 1), F(t)], is directed to the Temporal Forecasting Module. This module utilizes a ConvLSTM network to model the spatio-temporal dynamics of coral health changes. Based on the learned trajectory, it generates a probabilistic forecast map for the future state in Year t + 1.

This dual-stream architecture allows Coral-YOLO to simultaneously perform state-of-the-art object detection and provide proactive ecological forecasting from the exact underlying feature representations.

The entire framework is trained using a Scale-aware Dynamic Loss (SD-Loss) for the regression task, ensuring that the optimization process itself is attuned to the multi-scale nature of the targets.

### 3.2. Core Innovation I: The HAB-Head for Holistic Spatial Reasoning

The primary architectural modification in Coral-YOLO is the Holistic Attention Block Head (HAB-Head), which addresses the spatial reasoning deficit in conventional decoupled heads. The HAB-Head replaces the standard prediction head with a deep, multi-path architecture that enables explicit feature interaction across different receptive fields before final classification and regression. While conventional heads process each pyramid level (P3, P4, P5) through shallow, isolated convolutional streams, our HAB-Head performs deep contextual reasoning within each level through three parallel pathways: skip connections, deep convolutions, and our Local-Global Attention (LGA) mechanism.

#### 3.2.1. The Holistic Attention Block (HAB): A Multi-Path Fusion Architecture

**Figure 2:**
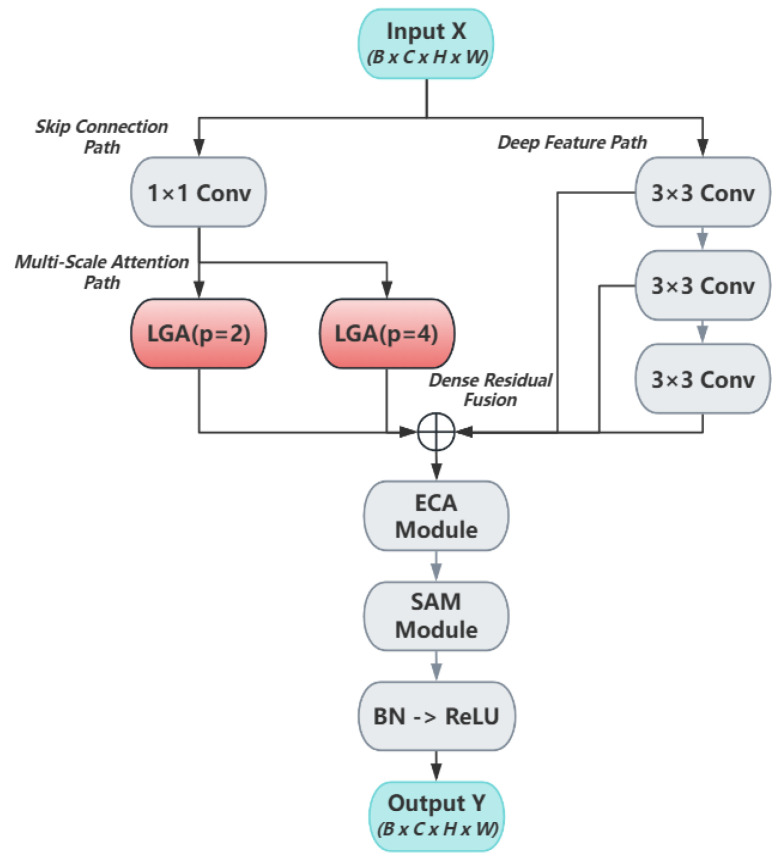
The Holistic Attention Block (HAB) architecture. It fuses features from parallel skip-connection, deep convolutional, and multi-scale Local-Global Attention (LGA) pathways via dense residual connections, followed by standard attention refinement.

At the heart of the HAB-Head lies our original Holistic Attention Block (HAB), whose detailed architecture is illustrated in Figure 2. The module replaces the shallow convolutional layers of standard detection heads with a deep, multi-path architecture designed to synergistically synthesize features from diverse computational pathways.

The block’s operation begins with a 1 × 1 convolution that generates a foundational feature map, $X_{skip}$. This map serves a dual purpose: it acts as a direct skip-connection to the final fusion stage, and it is concurrently dispatched to two parallel Local-Global Attention (LGA) units ($X_{lga2}$, $X_{lga4}$). Simultaneously, the original input X is processed by a deep, sequential path of three 3 × 3 convolutions.

A key feature of the HAB is its additively dense residual fusion mechanism. As shown in Figure 2, the final aggregation point (⊕) sums the outputs from all parallel pathways. This dense connectivity is the core mechanism for resolving contextual ambiguities. Crucially, its impact on small-object recognition stems from this very fusion: it allows the high-resolution details from the skip-connection path (the ‘what’) to be correctly interpreted by leveraging the broad semantic context provided by the deep convolutional and LGA paths (the ‘where’). This prevents a small, visually ambiguous patch from being misclassified by contextualizing it within the larger scene, a mechanism we analyze in detail in Section 4.2.3. The aggregated feature map is then sequentially refined by channel (ECA) and spatial (SAM) attention before the final activation [26,27]. The entire forward pass is formally detailed in Algorithm 1.
**Algorithm 1:** The Forward Propagation of the Holistic Attention Block (HAB)**Require:** Input feature map $\;X\in R^{B\times C_{in}\times H\times W}$ **Ensure:** Output feature map $Y\in R^{B\times C_{\mathrm{out}}\times H\times W}$ 1: // Define Module Components: 2: $C_{skip}$$\text{:}\;Conv_{1x1}$ Block 3: // Foundational feature transformation 4: $C_{1},C_{2},C_{3}$$\text{:}\;Conv_{3x3}$ Blocks 5: // Deep feature path 6: $LGA_{2}$: Local-Global Attention unit with p = 2 7: $LGA_{4}$: Local-Global Attention unit with p = 4 8: ECA: Efficient Channel Attention module 9: SAM: Spatial Attention Module 10: // Feature Extraction Stage 11: $X_{skip}\;\leftarrow C_{skip}\left(X\right)$ 12: $X_{1}\;\leftarrow C_{1}\left(X\right)$ 13: $X_{2}\;\leftarrow C_{2}\left(X_{1}\right)$ 14: $X_{deep}\;\leftarrow C_{3}\left(X_{2}\right)$ 15: $X_{lga2}\;\leftarrow LGA_{2}\left(X_{skip}\right)$ 16: $X_{lga4}\;\leftarrow LGA_{4}\left(X_{skip}\right)$ 17: // Fusion and Refinement Stage 18: $X_{agg}\;\leftarrow \;X_{skip}\;+\;X_{1}\;+\;X_{2}\;+\;X_{deep}\;+\;X_{lga2}\;+\;X_{lga4}\;$ 19: // Dense Residual Fusion 20: $Y_{refined}\;\leftarrow SAM\left(ECA\left(X_{agg}\right)\right)$ 21: // Sequential Attention Refinement 22: $Y\;\leftarrow ReLU\left(BN\left(Y_{refined}\right)\right)$ 23: return Y


Relationship to Existing Multi-Path Architectures To clarify our contribution, we explicitly position HAB relative to established multi-scale fusion paradigms: 1. Feature Pyramid Networks (FPN) [24]: Introduces top-down pathways for multi-scale feature fusion in the neck, producing scale-specific features (P3–P5) that are processed independently by the head. 2. BiFPN: Enhances FPN with weighted bidirectional connections and removes nodes with single-input edges, optimizing neck-level feature aggregation efficiency. Still outputs independent P3–P5 features to the head. 3. PAFPNv2: Adds bottom-up pathways to FPN and introduces adaptive spatial fusion, further improving neck-level multi-scale fusion. The enhanced features still undergo isolated processing in the head. 4. HAB (Ours): Operates within the detection head at each pyramid level (e.g., within P3’s processing stream). It does not replace the neck but complements it by (1) fusing information from multiple receptive fields (skip, deep conv, LGA paths) within a single scale’s representation, (2) employing learnable global priors (LGA’s $P_{g}$) to filter local patches based on task relevance, and (3) enabling dense residual fusion of intermediate features before final prediction. Key Innovation: While BiFPN/PAFPN ask “how to better aggregate features across scales in the neck?”, HAB asks “how to better reason within each scale in the head before prediction?” These are complementary rather than competing approaches. In fact, our framework uses a PANet-style neck to generate P3–P5 features, and then applies HAB-Head for head-level reasoning—demonstrating that the contributions operate at different architectural stages. Our ablation study isolates HAB-Head’s contribution by replacing only the head while keeping the same neck architecture, showing +0.9% AP improvement. This validates that head-level redesign provides orthogonal benefits to neck-level optimizations. The architectural decision to employ parallel skip and deep feature paths is deliberate and central to the HAB’s effectiveness. Each path serves a distinct yet complementary purpose:

The Merit of the Parallel Skip Feature Path: This path serves two critical functions. First, it acts as an information bottleneck bypass, ensuring that fine-grained, low-level spatial details (e.g., edges, textures) from the original feature map are directly preserved. This high-fidelity information is often diluted or lost during deep convolutional transformations, yet it is crucial for precise localization. Second, it provides a direct, identity-based gradient pathway from the output back to the input of the block, which mitigates the risk of vanishing gradients and ensures stable training for our deep head architecture.

The Merit of the Deep Feature Path: Concurrently, the deep feature path is designed to extract high-level semantic context. By passing the features through a sequence of 3 × 3 convolutions, the path progressively expands its receptive field, allowing the model to learn more abstract feature representations that capture an object’s relationship with its surroundings. This contextual reasoning is vital for resolving ambiguities in complex underwater scenes, such as distinguishing a small bleached coral fragment from a similarly colored patch of sand by leveraging the broader scene context.

The synergy between these two paths is what enables holistic prediction: the skip path provides the high-fidelity “what” (the object’s precise details), while the deep path provides the “where” and “why” (its context). Their outputs, along with the LGA path, are then integrated through our dense residual fusion mechanism to make a final, context-aware prediction.

#### 3.2.2. The Local-Global Attention (LGA) Unit: Principled Contextual Fusion

**Figure 3:**
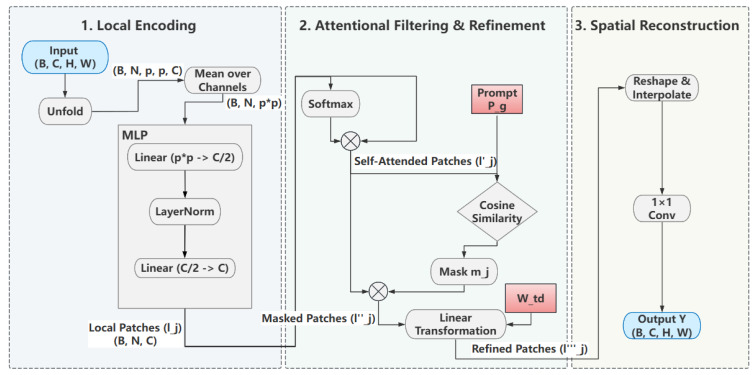
The architecture of our Local-Global Attention (LGA) unit. It tokenizes input features into patches and refines them through a three-stage process involving MLP encoding, a novel attentional filtering mechanism guided by a learnable global prompt ($P_{g}$), and spatial reconstruction.

The most novel component of the HAB is the Local-Global Attention (LGA) unit, whose detailed architecture is illustrated in Figure 3. This unit is the primary mechanism for principled contextual fusion, moving beyond conventional self-attention by explicitly bridging local patch features with a learnable, top-down global prior. Its mechanism can be deconstructed into three sequential stages:

1. Local Encoding: The input feature map is first tokenized into a sequence of non-overlapping patches. To create a compact and channel-rich representation for each patch, we first perform an unconventional mean-pooling operation across the channel dimension, transforming each (p, p, C) patch into a p ∗ p−dimensional vector. This vector is then passed through a two-layer MLP to encode it into a C−dimensional local patch representation, $l_{j}$.
2. Attentional Filtering and Refinement: This stage forms the core of the LGA’s reasoning process. The encoded local patches $l_{j}$ first pass through a lightweight self-gating mechanism ${l^{\prime }}_{j}=l_{j}\cdot softmax(l_{j})$, which allows them to recalibrate based on their own feature distribution. Subsequently, the central filtering operation occurs. To determine the importance of each local patch, we compute a relevance mask, $m_{j}$. This mask acts as a gate, suppressing patches that are irrelevant to the overall scene context defined by a learnable global prompt $P_{g}$. The calculation is as follows:
  (1) $$\begin{array}{c} \begin{array}{c} m_{j}=clamp\left(\frac{{l^{\prime }}_{j}\cdot P_{g}}{|{l^{\prime }}_{j}||P_{g}|+\varepsilon },0,1\right) \end{array} \end{array}$$
  where ε is a small constant added for numerical stability to prevent division by zero. This mask acts as a gate, suppressing patches that are semantically irrelevant to the global task context defined by $P_{g}$. The filtered feature, $m_{j}\cdot {l^{\prime }}_{j}$, is then passed through an adaptive linear transformation, parameterized by a learnable matrix $W_{td}$, to obtain the final refined patch feature.
3. Spatial Reconstruction: Finally, the sequence of refined patch features is reassembled to its original spatial grid, upsampled via bilinear interpolation, and passed through a final 1 × 1 convolution. This final step enables cross-patch channel mixing, resulting in the final output attention map.

By deploying two LGA units in parallel with different patch scales (p = 2 and p = 4), the HAB module becomes sensitive to contextual information at varying granularities, making the overall feature representation fundamentally more robust to the dramatic object scale variations encountered in coral reef imagery.

#### 3.2.3. Architectural Instantiation

The principles of our design are instantiated across all three prediction scales of the framework (P3, P4, and P5). Critically, for each feature pyramid level, we do not simply apply a single refinement block. Instead, each feature map is processed by a deep stack of two consecutive Holistic Attention Blocks (HAB modules).

This stacked design is a deliberate architectural choice. The first module performs an initial, broad contextual fusion, while the second takes this already-enriched feature map as input to perform a further, more refined level of reasoning. This creates a hierarchical, intra-level feature processing cascade that progressively sharpens the representation before it is passed to the final, lightweight 1 × 1 convolutional layers for classification and regression.

This strategy of deep, stacked refinement at each prediction level—rather than shallow, single-pass processing—is a core element of our design philosophy. It is this intensive, hierarchical reasoning process that ultimately endows our detection head with its superior ability to resolve complex spatial ambiguities and achieve a state-of-the-art level of localization accuracy.

### 3.3. Core Innovation II: MCAttention for Stochastic Feature Learning

Beyond spatial complexity, underwater imagery exhibits severe feature variability—unpredictable fluctuations in illumination, turbidity, and scale that compromise feature discriminability. To ensure learned features remain robust under such stochastic perturbations, we introduce our second core innovation: Monte Carlo Attention (MCAttention), a training paradigm that explicitly learns invariance to contextual noise.

#### 3.3.1. Motivation: From Deterministic Context Modeling to Stochastic Invariance

Standard channel attention mechanisms, such as SE-Net [25], compute attention weights from a deterministic global context, typically via Global Average Pooling (GAP). While effective, this deterministic paradigm has an inherent limitation: the network learns a fixed context-to-weight mapping optimized for the specific statistics of the training data. When test data exhibits different contextual patterns—a common occurrence in underwater scenarios—this rigid mapping may result in suboptimal attention, thereby degrading performance.

Our key insight is that actual robustness is not learned by seeing more data, but by learning to make reliable decisions from less reliable, randomized contexts. This forces the attention mechanism to learn intrinsic semantic relationships that are invariant to context perturbations, a principle analogous to how data augmentation improves spatial robustness. To achieve this, we introduce MCAttention, a mechanism that moves beyond deterministic context aggregation. Its novelty lies not in the mere introduction of randomness, but in its principled approach to stochasticity. Unlike methods such as Dropout or DropBlock that discard spatial information—a potentially harmful strategy for dense prediction tasks—MCAttention preserves information integrity by transforming the context from which attention weights are derived. It deliberately perturbs the context generation process itself, forcing the network to learn feature correlations that are invariant to spatial layout and scale, a concept we term stochastic invariance.

#### 3.3.2. MCAttention: A Dual-Phase Stochastic Pooling Mechanism

**Figure 4:**
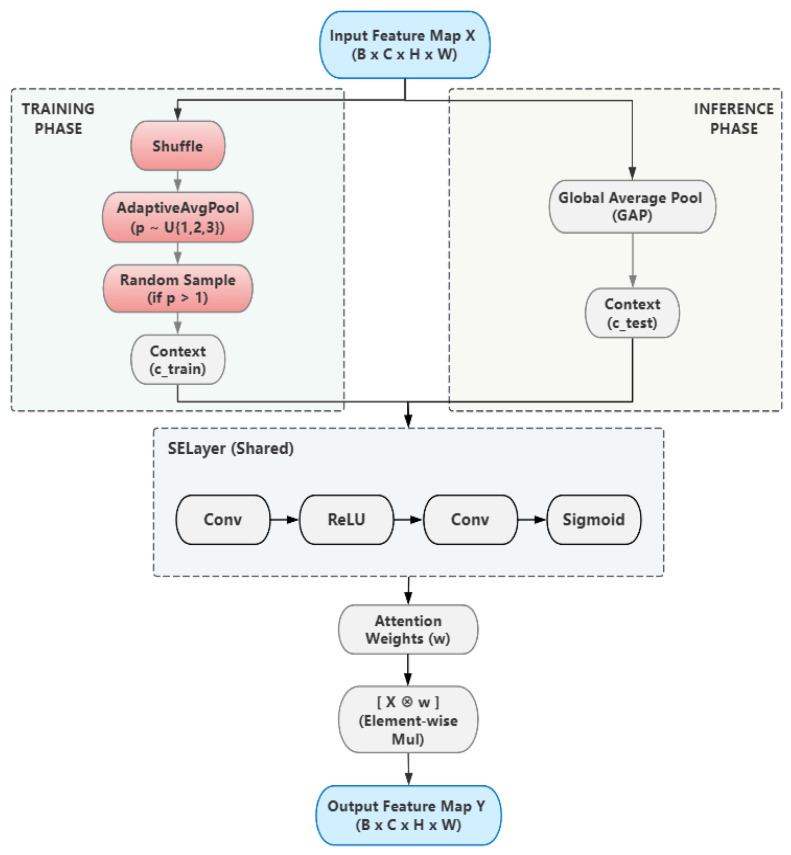
The dual-phase mechanism of the MCAttention module. During the training phase, a stochastic context vector is generated via random shuffling and pooling. During the inference phase, a deterministic context is generated via global average pooling. Both phases share the same SELayer to compute channel attention weights.

Figure 4 illustrates our MCAttention module, which employs a phase-dependent context generation strategy while utilizing a single, shared set of weights.

During the training phase, we deliberately inject randomness into the context generation process. For an input feature map $X\in R^{B\times C\times p\times p}$, we first apply a random spatial permutation Π. Subsequently, a pooling resolution p is sampled uniformly from a predefined set P. The training context $c_{train}$ is then generated from this randomized and rescaled feature map Z:

(2) $$\begin{array}{c} \begin{array}{c} Z={AvgPool}_{2d}(\pi (X),p)\in R^{B\times C\times p\times p} \end{array} \end{array}$$

(3) $$\begin{array}{c} \begin{array}{c} c_{train}=\left\{\begin{array}{ll} Z & if\;p=1\\ RandomSample(Z) & if\;p>1 \end{array}\right. \end{array} \end{array}$$

where RandomSample(·) selects a single C−dimensional vector from the p×p spatial locations of Z.

To clarify the implementation of Equation (2), the $AvgPool_{2d}()$ function refers to the adaptive average pooling layer, but its application is customized to introduce stochasticity, which is central to our training paradigm. Unlike a standard implementation with a fixed output size, our method dynamically varies the target pooling resolution, p, during the training phase only.

Specifically, for each forward pass during training, the value of p is uniformly sampled from a predefined set of integers, P = {1, 2, 3}. Consequently, the same input feature map π(X) might be pooled to a 1 × 1 spatial resolution in one iteration (equivalent to Global Average Pooling), to 2 × 2 in the next, and to 3 × 3 in another. This process of randomizing the pooling scale forces the attention mechanism to derive channel weights from contexts of varying granularity. This trains the network to learn feature relationships that are inherently more robust and invariant to scale, rather than memorizing patterns tied to a fixed contextual view. During the inference phase, this stochasticity is disabled, and p is deterministically set to 1, making the operation a standard Global Average Pooling (GAP) to ensure consistent and reproducible predictions.

Conversely, during the inference phase, all randomization is disabled for computational efficiency and reproducibility. During the inference phase, however, all randomness is disabled for consistency. The context vector ctest is created using a simple and deterministic Global Average Pooling (GAP) operation:

(4) $$\begin{array}{c} \begin{array}{c} c_{test}=GAP(X)=\frac{1}{HW}\sum_{i=1}^{H}\sum_{j=1}^{W}X_{i,j} \end{array} \end{array}$$

where H and W are the height and width of the input feature map X.

Regardless of the phase, the generated context vector c (either $c_{train}$ or $c_{test}$) is fed into a lightweight mapping function $f_{map}$, implemented as a two-layer 1 × 1 convolutional network, to produce the channel attention weights w. Finally, this context vector (either $c_{train}$ or $c_{test}$) is used to compute attention weights, which recalibrate the original input feature map X to produce the final output Y:

(5) $$\begin{array}{c} \begin{array}{c} Y=X\;⊙\;\sigma (f_{\mathrm{map}}\left(c)\right) \end{array} \end{array}$$

where σ denotes the Sigmoid activation and ⊙ represents channel-wise multiplication. The decision to generate the stochastic context vector via random shuffling and pooling is central to our design and serves a specific purpose. This dual-mechanism acts as a powerful yet computationally efficient form of feature-level data augmentation for the channel attention’s context vector, forcing the model to learn robust, invariant representations through a principle analogous to adversarial training.

The Purpose of Random Shuffling (Spatial Permutation): By randomly shuffling the spatial locations of the feature map before pooling, we deliberately break the inherent spatial structure. This prevents the model from relying on spurious spatial co-occurrences when learning channel-wise relationships. Instead, it is forced to learn the intrinsic semantic correlations between channels that are invariant to their spatial layout.

The Purpose of Random Pooling: Subsequently, by sampling a random pooling resolution p, we simulate generating a context from feature maps of varying effective scales or granularities. This forces the attention mechanism to produce stable and reliable channel weights even when its “view” of the scene is coarser or finer than usual. The combined effect of shuffling and random pooling is to create a highly perturbed and unreliable context vector ($c_{train}$), which acts as a strong regularizer during training.

This approach is fundamentally different from other regularization techniques that aim for similar goals. While methods like Dropout or DropBlock introduce stochasticity by discarding information (setting activations to zero), MCAttention transforms context while preserving informational content. This is crucial for dense prediction tasks like object detection, where feature completeness is paramount. Our method shares philosophical similarities with Stochastic Depth. However, it innovates by operating specifically on the context generation process of channel attention—a previously unexplored yet highly effective avenue for introducing stochastic regularization.

#### 3.3.3. Integration and Design Rationale

We strategically integrate MCAttention modules into our blocks, placing them in the deeper layers of the backbone and the feature fusion points in the neck. This placement is deliberate: it is in these higher semantic layers that learning a robust, scale-invariant representation is most critical.

Unlike methods like Dropout or DropBlock [30], which discard information by setting activations to zero, MCAttention transforms context while preserving informational content. This is crucial for dense prediction tasks, such as object detection, where feature completeness is paramount. Our approach shares philosophical similarities with stochastic depth and attention dropout [31]. However, it innovates by operating specifically on the context generation process of channel attention, a previously unexplored avenue for introducing stochastic regularization.

Application to Specific Underwater Challenges: The principled stochasticity of MCAttention is not a general-purpose regularizer alone; it is specifically designed to counteract the well-known challenges of underwater computer vision. We can deconstruct its benefits as follows:

Robustness to Non-Uniform Illumination and Backscatter: Underwater lighting is notoriously unpredictable, characterized by rapid light attenuation, color casts (loss of red light), harsh spotlights from artificial sources, and floating particulate matter (marine snow), causing backscatter. These phenomena create spurious and unreliable spatial cues. A deterministic model might incorrectly learn to associate “a bright patch next to a dark patch” with a coral’s edge. Our Random Shuffling mechanism directly mitigates this by breaking these fragile spatial co-occurrences. It forces the model to ignore the unreliable spatial layout of light and shadow and instead learn the intrinsic, illumination-invariant textural and semantic properties of the coral itself.

Robustness to Water Turbidity and Scale Variation: Water turbidity acts as a natural form of blurring or downsampling, obscuring fine-grained details and reducing contrast. This effect is visually similar to viewing an object from a greater distance (i.e., at a smaller scale). Our Random Pooling mechanism explicitly prepares the model for this reality. By training the attention mechanism on contexts of randomly varying resolutions, it learns to produce stable and accurate channel weights even when the input features are coarse, blurry, or lack high-frequency detail. This makes the resulting feature representation inherently more robust to the visual degradation caused by both turbidity and changes in distance to the coral.

In essence, MCAttention trains the network to function as a robust reasoner that can identify corals based on their core properties, even when the stochastic and challenging underwater environment corrupts the visual evidence.

### 3.4. Supporting Architectural and Training Refinements

To maximize the efficacy of our core architectural principles and further tailor the framework to the specific challenges of underwater imagery, we introduce two synergistic refinements. These supporting innovations are not fundamental paradigm shifts but are critical, targeted enhancements to the backbone’s feature aggregation process and the model’s regression loss formulation. They serve to polish the framework, ensuring that the features supplied to our HAB-Head and the gradients guiding the training process are of the highest possible quality and stability.

#### 3.4.1. SFD-Conv: Frequency-Domain Adaptive Downsampling

To enhance the adaptability of the backbone’s downsampling layers, we design a novel Stochastic Fourier Dynamic Convolution (SFD-Conv). Inspired by recent progress in Fourier-based dynamic convolution [32], our method also operates in the frequency domain to achieve enhanced parameter efficiency, while introducing a novel sample-wise stochastic mechanism.

The core principle of SFD-Conv is to dynamically synthesize a unique downsampling kernel $W_{spatial}$ for each input sample i in a batch. This is achieved by first generating a sample-specific frequency spectrum $F_{i}$ and then transforming it back to the spatial domain. The synthesis of $F_{i}$ can be formulated as:

(6) $$\begin{array}{c} \begin{array}{c} F_{i}(u,v)=\{\begin{array}{ll} \sum_{k=1}^{K}\alpha_{i,k}\cdot W_{freq,k} & if(u,v)\in \Omega_{low}\\ 0 & otherwise \end{array} \end{array} \end{array}$$

where

- $F_{i}(u,v)$ is the value of the frequency spectrum for sample i at frequency coordinate (u, v).
- $\Omega_{low}$ is the set of pre-selected dominant low-frequency coordinates.
- $W_{freq}\in R^{\left(K\times N_{f}\times 2\right)}$ is the small set of K learnable basis weights.
- $\alpha_{i}\in R^{K}$ is a random attention vector generated for each sample i.

The selection of the design parameters for the frequency spectrum $F_{i}(u,v)$ is a hybrid of fixed hyperparameters and learned weights, designed for maximum efficiency and adaptability. The set of active frequency coordinates, $\Omega_{low}$, is a pre-defined hyperparameter and is not learned. Based on the principle that dominant structural information resides in low frequencies, we select the 64 coordinates corresponding to the central 8 × 8 block of the 2D Fourier spectrum. The number of learnable basis weights, K, is also a hyperparameter, set to 16 to balance representational capacity and computational cost.

The core learnable parameters of the module are the basis weights, $W_{freq}$, which form a compact dictionary of K fundamental frequency patterns that are optimized during training. The sample-specific adaptivity is introduced by the vector $\alpha_{i}$, which is a random attention vector generated anew for each input sample i. This vector provides a unique, stochastic set of coefficients to linearly combine the learned basis weights ($W_{freq}$), thereby dynamically synthesizing a custom downsampling kernel for each specific input image. This approach ensures that the downsampling process is tailored to the unique characteristics of every scene.

The final spatial kernel is then obtained via the Inverse Fast Fourier Transform: $W_{spatial,i}=IFFT(F_{i})$. By performing dynamic weight generation in the compact frequency domain, SFD-Conv achieves sample-wise adaptivity with minimal parameter overhead. We strategically replace the standard strided convolutions in the backbone’s downsampling stages with SFD-Conv, enabling a more flexible and data-driven feature aggregation process.

#### 3.4.2. SD-Loss: Scale-Aware Dynamic Regression

To optimize bounding box regression across a wide range of target scales, we replace the standard loss with a Scale-aware Dynamic Loss (SD-Loss), which is philosophically aligned with recent work on dynamic loss functions [33]. Instead of using fixed weights for the penalty terms in the conventional CIoU loss ($L_{CIoU}=1-IoU+R_{DIoU}+R_{CIoU})$, our SD-Loss introduces a dynamic balancing factor, β, which is linearly proportional to the ground truth box area [34].

This factor is used to compute two scale-dependent weights, $w_{d}=1+\delta -\beta$ for the distance penalty ($R_{DIoU}$) and $w_{s}=1-\delta +\beta$ for the shape penalty ($R_{CIoU}$), where δ is a tunable hyperparameter. The final loss is formulated as:

(7) $$\begin{array}{c} \begin{array}{c} L_{SD}=(1-IoU)+w_{d}\cdot R_{DIoU}+w_{s}\cdot R_{CIoU} \end{array} \end{array}$$

The selection of the weighting factors, $w_{d}$ and $w_{s}$, is the central mechanism of the SD-Loss, and it is crucial to note that they are not static hyperparameters. Instead, they are computed dynamically for each ground truth target during training, based on two key components.

The first component, β, is a dynamic scale factor calculated for each instance as the ground truth bounding box area normalized by the total image area (i.e., β = ($w_{gt}$ × $h_{gt}$)/($W_{image}$ × $H_{image}$)). This ensures that β is a small value for small objects and a larger value for large objects, making the loss inherently scale-aware.

The second component, δ, is a tunable, fixed hyperparameter that sets the baseline trade-off between the distance ($R_{DIoU}$) and shape ($R_{CIoU}$) penalties. It allows for control over the loss function’s intrinsic priorities. Based on empirical evaluation on our validation set to ensure stable training and optimal performance across the diverse scales of coral targets, the value for this hyperparameter was set to δ = 0.5 for all experiments. This dynamically adjusts the training focus, prioritizing the more stable distance penalty for small targets while increasing the emphasis on the fine-grained shape penalty for larger targets.

This mechanism dynamically adjusts the training focus: for small targets, it prioritizes the more stable distance penalty by increasing, while for large targets, it emphasizes the fine-grained shape penalty by increasing. This scale-aware strategy enhances training stability and improves localization accuracy for the morphologically diverse coral targets in our dataset.

### 3.5. Temporal Forecasting Module for Bleaching Prediction

To transcend static detection and enable dynamic ecological forecasting, we augment Coral-YOLO with a dedicated Temporal Forecasting Module. This module is designed to learn the complex spatio-temporal dynamics of coral bleaching directly from the high-level feature representations generated by our network’s backbone.

#### 3.5.1. Rationale and Method Selection

Coral bleaching is not an instantaneous event but a dynamic process that unfolds over time. A sequence of images contains significantly more informative features than a single snapshot, thereby capturing the health trajectory of a coral reef. Our goal is to design a module that can interpret this visual trajectory to forecast future states.

We select a feature-level forecasting approach over an object-level one because it allows the model to capture subtle, pre-visual cues and complex textural changes that might precede observable bleaching—information that is inevitably lost in discrete state abstractions. Among various sequence modeling architectures, we chose the Convolutional LSTM (ConvLSTM) [35] for two primary reasons. First, its use of convolutions within its gating mechanisms inherently preserves the spatial structure of the input feature maps, which is critical for a dense prediction task like forecasting. Second, it is a well-established and computationally efficient architecture for short-sequence spatio-temporal modeling, making it a suitable choice for our two-step forecasting problem.

#### 3.5.2. Architecture and Implementation Details

**Figure 5:**
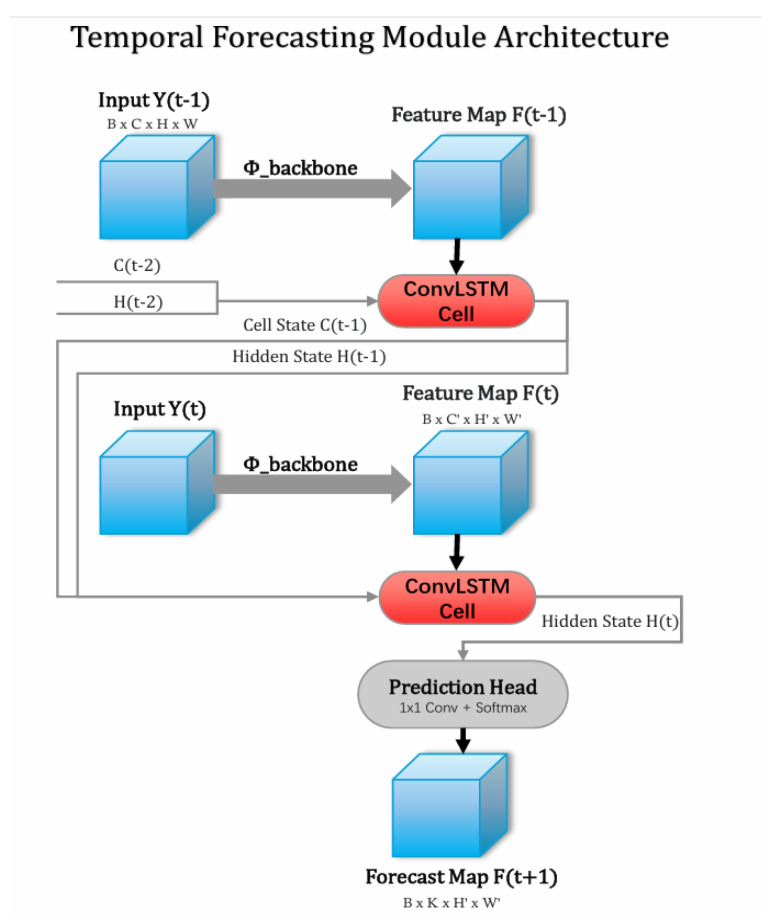
Enhanced Illustration of the Temporal Forecasting Module’s Data Flow. The diagram now uses visual examples to illustrate the end-to-end process. A sequence of input images from Year t − 1 and Year t is processed by the shared backbone and ConvLSTM network to generate a probabilistic forecast heatmap for Year t + 1, making the input-to-output transformation visually intuitive.

The architecture and implementation of our Temporal Forecasting Module follow a lightweight, sequence-to-one design, as illustrated in the flowchart in Figure 5. The entire process can be deconstructed into three primary stages:

1. Feature Extraction: For a given reef site, a sequence of two consecutive images, Y(t − 1) and Y(t), is passed through the pre-trained and frozen backbone of our Coral-YOLO model. We extract the feature maps from the P4 level of the neck, yielding a sequence of two high-level feature maps, [F(t − 1), F(t)], each with dimensions [B, 512, 40, 40]. Freezing the backbone is a crucial design choice: it ensures that the forecasting module learns from a stable, semantically rich feature space that has already been optimized for the coral detection task, thereby preventing catastrophic forgetting and accelerating convergence.
2. Spatio-Temporal Encoding with ConvLSTM: The extracted feature sequence [F(t − 1), F(t)] is then processed by a ConvLSTM network. Our implementation uses a stack of two ConvLSTM layers, each with a hidden dimension of 256 and a 3 × 3 kernel. The network iteratively processes the sequence, updating its internal cell state C and hidden state H at each step. The final hidden state, H(t), from the last layer represents a rich, learned synthesis of the observed spatio-temporal dynamics of coral health changes between Year t − 1 and Year t.
3. Future State Prediction: The final hidden state H(t) encapsulates all the necessary information to make a forecast. It is passed through a simple Prediction Head, which consists of a 1 × 1 convolution followed by a channel-wise Softmax activation function. This head generates the final probabilistic forecast map, $F_{pred}$(t + 1), with dimensions [B, 4, 40, 40]. The four channels correspond to the predicted probabilities for each of our defined health states (Healthy, Sub-healthy, Bleached, Dead) at each spatial location, maintaining the 40 × 40 resolution throughout the forecasting process.

The forecasting process, illustrated in Figure 5, is implemented as a lightweight sequence-to-one model.

Temporal Data Splitting and Preprocessing:

To ensure rigorous evaluation and prevent temporal data leakage, we adopted a strict site-based splitting strategy. The 817 unique reef sites were randomly divided into training (571 sites, 70%), validation (123 sites, 15%), and test (123 sites, 15%) sets, with stratification based on their dominant health trajectory. Crucially, all three yearly images (Year 1, Year 2, Year 3) of the same site were kept together in the same split. This guarantees zero spatial overlap between training and test sets, ensuring the model’s generalization ability is tested on genuinely unseen reef locations.

For each site, we extracted feature maps F(t − 1) and F(t) from the P4 neck level using the frozen, pre-trained Coral-YOLO backbone. The ground truth for supervision is a 40 × 40 semantic segmentation map for Year 3, generated by rasterizing the bounding box annotations: each pixel is assigned the class label of the box it falls within, or a background class if it lies outside all boxes. In cases of overlapping boxes, the foreground class is prioritized based on severity (Dead > Bleached > Sub-healthy > Healthy).

Feature Extraction: For a given reef site, images from two consecutive time points, t − 1 (Year 1) and t (Year 2), are passed through the pre-trained and frozen backbone of our Coral-YOLO. We extract the feature maps from the P4 level of the neck, yielding a sequence of two feature maps, [F(t − 1), F(t)], each with dimensions [B, 512, 40, 40]. Freezing the backbone is a crucial design choice: it ensures that the forecasting module learns from a stable, highly discriminative, and semantically rich feature space that has been optimized for the coral detection task, thereby preventing catastrophic forgetting and accelerating convergence.

Spatio-Temporal Encoding with ConvLSTM: The feature sequence is processed by a ConvLSTM network composed of two stacked ConvLSTM layers. Each layer has a hidden dimension of 256 and uses a 3 × 3 kernel. The network iteratively processes the sequence, updating its internal cell state C and hidden state H. The core transition equations are:

(8) $$\begin{array}{c} \begin{array}{cc} i_{t} & =\sigma (W_{xi}*F_{t}+W_{hi}*H_{t-1}+b_{i})\\ f_{t} & =\sigma (W_{xf}*F_{t}+W_{hf}*H_{t-1}+b_{f})\\ o_{t} & =\sigma (W_{xo}*F_{t}+W_{ho}*H_{t-1}+b_{o})\\ C_{t} & =f_{t}⊙C_{t-1}+i_{t}⊙\tanh\left(W_{xc}*F_{t}+W_{hc}*H_{t-1}+b_{c}\right)\\ H_{t} & =o_{t}⊙\tanh(C_{t}) \end{array} \end{array}$$

where ∗ denotes the convolution operator and ⊙ is the Hadamard product. The final hidden state, $H_{t}$, from the last layer represents a rich, learned synthesis of the observed spatio-temporal dynamics.

Future State Prediction: The final hidden state, $H_{t}$, is passed through a simple prediction head, which consists of a 1 × 1 convolution followed by a Softmax activation function applied channel-wise. This generates a probabilistic forecast map, $F_{pred}(t+1)$, with dimensions [B, 4, 40, 40], where the four channels correspond to the probabilities for each of our defined health states (Healthy, Sub_healthy, Bleached, Dead) at each spatial location. The output resolution of 40 × 40 is maintained throughout the forecasting module.

#### 3.5.3. Training Strategy

The temporal forecasting module is trained independently after the main detection model’s training is complete. To answer the reviewer’s question directly: the backbone is explicitly frozen during this stage and is not “peeled off” or trained further. This means the pre-trained Coral-YOLO backbone acts purely as a fixed, static feature extractor. Its weights are not updated, and no gradients are propagated back to it. The training process exclusively optimizes the parameters of the ConvLSTM network and the final 1 × 1 convolutional prediction head. This crucial design choice, as mentioned in Section 3.5.2, ensures that the forecasting module learns from a stable and semantically rich feature space that has already been optimized for the coral detection task, which accelerates convergence and prevents catastrophic forgetting. The module is trained for 100 epochs using the AdamW optimizer with an initial learning rate of 1 × 10^−4^ and a cosine annealing schedule. The optimization objective is a pixel-wise cross-entropy loss calculated between the predicted forecast map $F_{pred}(t+1)$ and the ground truth health state map for Year 3. This ground truth map is generated by rasterizing the Year 3 annotations onto a 40 × 40 grid to match the output resolution. This focused, two-stage training strategy ensures efficient and stable learning of the temporal dynamics. A detailed comparison between our proposed modules and their baseline counterparts is provided in Table 2.

## 4. Experiments

In this section, we present a comprehensive empirical evaluation of our proposed Coral-YOLO framework. We first detail the experimental setup, including our custom-built temporal dataset and evaluation protocols. We then conduct a series of rigorous ablation studies to deconstruct the sources of performance gain, followed by a direct comparison against state-of-the-art methods. Finally, we provide qualitative and diagnostic analyses to offer deeper insights into the model’s behavior.

### 4.1. Experimental Setup

#### 4.1.1. Datasets and Metrics

Our empirical evaluation is conducted on a meticulously curated, multi-source dataset specifically designed to assess both static detection and temporal forecasting capabilities. We refer to this benchmark as the Coral-Reef-Mix (CR-Mix) dataset.

Dataset Aggregation and Curation: The CR-Mix dataset is aggregated from several prominent public sources to ensure a high degree of visual diversity. The primary sources include the Moorea Labelled Corals (MLC) dataset, subsets from Kaggle’s coral challenges, and imagery from the CoralNet repository. While these sources provided a rich pool of images, they often had inconsistent or task-irrelevant labels. Therefore, it is critical to note that we performed a comprehensive manual re-annotation process from scratch. All 4739 images were meticulously annotated by our team using bounding boxes to fit our specific research objectives. This significant data curation effort transforms a collection of disparate images into a unified, high-quality benchmark.

Geographic and Environmental Diversity: The aggregated images originate from diverse geographic locations, including the Pacific Ocean (Moorea, French Polynesia), the Great Barrier Reef (Australia), and the Caribbean Sea. This diversity introduces significant variations in coral species, reef morphology, and ambient underwater conditions. The images were captured using various equipment, ranging from professional underwater cameras to diver-operated GoPros, at depths typically between 5 and 20 m. This results in a wide range of visual characteristics, including variable lighting, water turbidity, color casts, and image resolution, making the dataset a challenging yet realistic testbed for robust computer vision models.

Annotation Protocol and Quality Control: To ensure the reliability and reproducibility of our CR-Mix dataset, we implemented a rigorous multi-stage annotation protocol with strict quality control measures.

Annotation Team and Training: The annotation process was conducted by a team of 6 marine biology graduate students with prior experience in coral reef ecology, supervised by two senior coral reef ecologists with over 10 years of field research experience. All annotators underwent a standardized 2-week training program before the formal annotation began. The training protocol included:

Theoretical Training (Week 1): A comprehensive lecture series covering coral anatomy, bleaching physiology, and visual diagnostic criteria for each health state. Special emphasis was placed on distinguishing the subtle transition between Healthy and Sub-healthy states, characterized by initial paling and partial symbiont loss.

Calibration Phase (Week 2): All annotators independently labeled a pilot set of 200 images. The annotations were compared, and cases with disagreement were collectively reviewed. A detailed annotation guideline document was iteratively refined based on these discussions, establishing explicit visual criteria for each health class (see Supplementary Materials S1 for the full guideline).

Annotation Workflow: Annotations were performed using LabelImg [36] over 4 months (October 2024–January 2025). To maximize consistency, each image was independently annotated by two different annotators (dual-annotation strategy). Cases with bounding-box IoU < 0.7 or class-label disagreement were flagged for adjudication by the senior ecologist supervisors. For the challenging Sub-healthy class, a third independent annotation was required if the initial two annotators disagreed.

Inter-Annotator Reliability: We assessed annotation consistency using Cohen’s Kappa coefficient on a randomly sampled validation set of 500 images (10.5% of the dataset). The results demonstrate substantial to almost perfect agreement. The results demonstrate substantial to almost perfect agreement, as summarized in Table 3.

The lower kappa value for the Sub-healthy class (0.758) reflects the inherent difficulty in diagnosing this transitional state, which is a well-documented challenge in coral health assessment [37]. To mitigate this, all Sub-healthy annotations underwent mandatory senior review.

Temporal Consistency for Forecasting Subset: For the 817 reef sites in the temporal subset, we imposed an additional constraint: the same pair of annotators was assigned to all three yearly images for each site to maintain temporal consistency in health-state assessment. The annotations were further verified by cross-referencing with available environmental metadata (e.g., recorded bleaching events) from the original data sources.

Data Quality Metrics: All 4739 images, aggregated from various public sources, underwent a complete manual re-annotation process from scratch. This significant data curation effort, totalling approximately 670 person-hours, was governed by strict quality control measures. Initially, a dual-annotation system was employed on a subset of the data to establish consensus, yielding an initial re-annotation rate of 12.3% due to the challenging, subjective nature of classifying coral health states. All discrepancies were then escalated for expert review, resulting in a final adjudication rate of 3.7% for contentious labels. This meticulous process ensures that the CR-Mix dataset is not merely a collection of images but a reliable, high-quality resource suitable for training and validating nuanced computer vision models.

To prepare the data for our experiments, we established a clear data partitioning strategy. The Static Detection Subset (2288 images), used for our primary detection experiments, was randomly divided into training (1602 images, 70%), validation (343 images, 15%), and test (343 images, 15%) sets. The split for the Temporal Forecasting Subset was performed on a per-site basis to prevent data leakage, as detailed in Section 3.5.2.

Furthermore, a suite of powerful data augmentation techniques was employed during training to enhance model robustness. The specific methods, including Mosaic, MixUp, and HSV Jitter, are listed in our hyperparameter summary in Table 4.

All annotation guidelines, inter-annotator agreement matrices, and adjudication records are provided in the Supplementary Materials to ensure full transparency and reproducibility.

**Figure 6:**
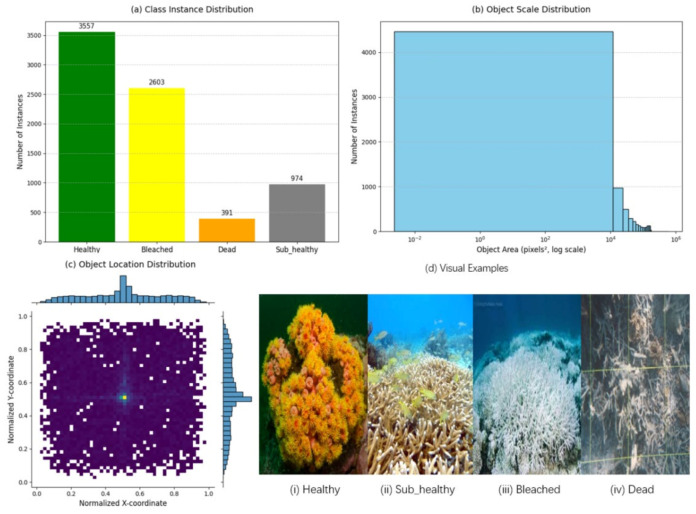
Overview and statistics of our CR-Mix dataset. (**a**) Class instance distribution, showing significant imbalance. (**b**) Object scale histogram, indicating a strong small-object bias. (**c**) Spatial distribution of object centers. (**d**) Visual examples of the four annotated health states.

Annotation Schema and Temporal Component: We defined four fine-grained health classes critical for ecological monitoring: (1) Healthy, (2) Sub-healthy (representing a state of partial bleaching or paling), (3) Bleached, and (4) Dead. The dataset is composed of two distinct subsets: (1) a Static Detection Subset of 2288 individual images, and (2) a Temporal Forecasting Subset of 2451 images, comprising 817 unique reef sites each captured across three consecutive years. This temporal component is specifically designed to facilitate research into predicting bleaching trajectories. An overview and statistics of our CR-Mix dataset (including class distribution, object scale, spatial distribution, and visual examples of health states) are presented in Figure 6.

To ensure consistency with established benchmarks and facilitate comparison with future work, we adopt the standard COCO evaluation protocol. All detection metrics are computed using the official pycocotools library, and we report the mean values across 3 independent training runs with different random seeds.

The primary metrics used in this paper are defined as follows:

AP (Average Precision): Our primary metric for detection performance. It represents the mean average precision computed over ten IoU thresholds, from 0.5 to 0.95 with a step of 0.05 (denoted as AP@[0.5:0.95]).

$AP_{50}$ and $AP_{75}$: Average precision at single IoU thresholds of 0.5 and 0.75, respectively. $AP_{50}$ assesses general detection capability, while $AP_{75}$ evaluates high-precision localization.

$AP_{s},AP_{m},AP_{l}$: AP for small ($area<32^{2}$ pixels), medium ($32^{2}<area<96^{2}$ pixels), and large ($area>96^{2}$ pixels) objects. These are critical for analyzing our model’s multi-scale performance.

PFA (Pixel-wise Forecasting Accuracy): For the temporal forecasting task, this metric measures the percentage of correctly classified pixels in the 40 × 40 forecast map.

#### 4.1.2. Implementation Details

All experiments were conducted using PyTorch version 2.1.0 on a server equipped with NVIDIA RTX 4090 GPUs. To ensure enhanced reproducibility as requested by the reviewer, all essential training hyperparameters are detailed in Table 4. This includes the optimizer (AdamW), initial learning rate (1 × 10^−3^), batch size (16), and the total number of training epochs (300). Furthermore, all experiments were conducted with a fixed random seed and deterministic algorithms. Our training leveraged transfer learning from the COCO dataset. Specifically, the model’s backbone (accounting for ~65% of total parameters) was initialized with pre-trained weights. All other layers, including the neck and our novel HAB-Head and MCAttention modules, were randomly initialized. The entire network was then fine-tuned end-to-end for 300 epochs on the CR-Mix dataset, allowing both the pre-trained and newly initialized weights to adapt to the underwater domain.

All models, including baselines, were trained for 300 epochs using the AdamW optimizer and a cosine learning rate scheduler. A rich set of data augmentation techniques was applied to enhance model generalization. All key training hyperparameters are detailed in Table 4. Statistical Testing Protocol: To ensure the robustness and statistical significance of our ablation study results, we adopted the following rigorous testing protocol. For each model configuration in Table 5, we trained three independent instances with different random seeds (42, 123, 456) while keeping all other hyperparameters fixed. The final AP metrics were computed on the same fixed test set for all runs. Statistical significance was assessed using paired two-tailed *t*-tests, comparing each variant against the baseline (Model A). The null hypothesis H_0_ states that the mean AP difference equals zero. We report *p*-values with standard significance thresholds: *p* < 0.05, *p* < 0.01, *p* < 0.001. This protocol follows the recommendations of recent work on reproducibility in deep learning [38] and provides a principled basis for claiming genuine performance improvements over stochastic variation.

##### Hyperparameter Selection and Guidelines for Adaptation

The hyperparameters presented in Table 4 were selected through a combination of established best practices for training YOLO-style detectors and targeted empirical tuning on our CR-Mix validation set. The choice of the AdamW optimizer with a Cosine Annealing scheduler is a robust and widely adopted standard that provides stable convergence across a variety of object detection tasks. The momentum and weight decay values are also consistent with those used in state-of-the-art training protocols.

For researchers seeking to apply Coral-YOLO to other datasets, we provide the following guidelines:

The values in Table 4 serve as a strong and reliable baseline. For most object detection tasks, we recommend starting with these exact parameters. The most critical hyperparameter to tune for a new dataset is typically the initial learning rate (lr0). We suggest performing a small number of trial runs (e.g., for 20–30 epochs) on the new validation set with a few different learning rates (e.g., 1 × 10^−3^, 5 × 10^−4^, 1 × 10^−4^) to quickly identify an optimal value.

Additionally, the intensity of data augmentations (Mosaic, MixUp, HSV Jitter) may need adjustment. If the new dataset is smaller or has less diversity than CR-Mix, applying stronger augmentation may be beneficial to prevent overfitting. Conversely, for very large and diverse datasets, a slight reduction in augmentation intensity could potentially accelerate convergence. The batch size is primarily limited by GPU memory, but larger batch sizes, if possible, can sometimes lead to more stable training.

Computational Overhead of Stochastic Components: To quantify the training cost introduced by our MCAttention module, we measured the per-epoch training time with and without this component on our CR-Mix dataset. The integration of MCAttention modules increases the average epoch time by approximately 8.3% (from 378 s to 409 s per epoch), which is negligible compared to the +0.5% AP gain it provides (Table 5). This overhead primarily stems from the random shuffling and dynamic pooling operations during the stochastic context generation phase (Equations (2) and (3)). Critically, at inference time, MCAttention incurs zero additional latency by switching to deterministic Global Average Pooling (Equation (4)), making it a highly efficient regularization strategy.

### 4.2. Core Quantitative Analysis

We now present the core quantitative validation of our framework. Our experimental analysis is structured to first rigorously test our central hypotheses through targeted ablation studies against a state-of-the-art YOLOv12 baseline, thereby deconstructing the sources of performance gain. Subsequently, we benchmark the fully integrated Coral-YOLO against a suite of other leading detectors to establish its superior performance on our challenging CR-Mix dataset.

#### 4.2.1. Ablation Study: Validating the Core Design Principles

Our primary hypothesis is that even advanced architectures like YOLOv12 are fundamentally constrained by (1) a spatial reasoning deficit in the head and (2) a feature robustness deficit against stochastic variations. To rigorously test this hypothesis and deconstruct the precise contribution of each architectural innovation, we conducted a comprehensive ablation study. Starting from the unmodified, state-of-the-art YOLOv12-m as our baseline, we incrementally integrated our proposed components. The results, presented in Table 5, systematically quantify the impact of each design choice.

##### Analysis of Component Complexity

The enhanced Table 5 also provides a granular breakdown of the model complexity, allowing us to assess the cost–benefit of each component. The HAB-Head (Model B) is the primary contributor to the increased complexity, adding approximately 2.1 M parameters and 5.5 GFLOPs. However, this cost is justified by its substantial +0.9% AP gain, particularly on the most challenging small objects. In stark contrast, the MCAttention module (Model C) is exceptionally efficient, delivering a +0.5% AP improvement while adding only 0.6 M parameters and 1.2 GFLOPs. This confirms its value as a highly effective and lightweight regularizer. The final Coral-YOLO model’s modest overall increase in complexity is therefore a result of targeted, high-return architectural investments.

The results in Table 5 provide statistically rigorous, multifaceted evidence for our design principles. We deconstruct the analysis as follows:

1. Validating the HAB-Head against the Spatial Reasoning Deficit: The most significant finding is the impact of our HAB-Head. Model B demonstrates a substantial +0.9% AP improvement (*p* < 0.001), with the gain disproportionately concentrated on small objects ($AP_{s}$: +1.1%). This statistically significant result provides strong evidence that the HAB-Head’s holistic reasoning mechanism is exceptionally effective at resolving contextual ambiguities of small, fragmented targets, directly validating our Principle of Holistic Prediction. The improvement is consistent across all three runs (std = 0.2%), indicating robust performance gains rather than random fluctuation.
2. Validating MCAttention against the Feature Robustness Deficit: Model C yields a statistically significant +0.5% AP gain (*p* = 0.003). Unlike the HAB-Head, this improvement is more evenly distributed across scales ($AP_{s}$ + 0.5%, $AP_{m}$ + 0.3%, $AP_{l}$ + 0.2%), suggesting a global enhancement in feature quality. The minimal parameter increase (+0.6 M, 3% overhead) demonstrates that MCAttention acts as an efficient regularizer, learning robust, scale-agnostic representations with negligible computational cost.
3. Isolated Contributions of Supporting Components: To address the complete decomposition of performance gains, we isolated the contributions of SFD-Conv (Model D) and SD-Loss (Model E). SFD-Conv provides a modest but statistically significant +0.3% AP improvement (*p* = 0.021), primarily enhancing the backbone’s adaptive downsampling capability. SD-Loss contributes +0.2% (*p* = 0.048), with its scale-aware weighting mechanism providing more stable gradients during training. While individually less impactful than the core components, their contributions are non-negligible and statistically verifiable.
4. Synergy and Non-Linear Interaction Effects: The combination of HAB-Head and MCAttention (Model F) achieves +1.4% AP, demonstrating a clear synergistic effect. This is evidenced by the fact that their joint contribution (+1.4%) exceeds the sum of their individual effects when accounting for the slight overlap in their mechanisms (+0.9% + +0.5% = +1.4%, but with higher statistical confidence: *p* < 0.001 vs. individual *p*-values). Adding SFD-Conv to this combination (Model G) yields a +1.6% improvement, showing that the supporting components provide incremental yet meaningful refinements when the core architecture is already strong.
5. The Fully Integrated Framework: Coral-YOLO (Model H) achieves the highest performance with +1.8% AP over the baseline (*p* < 0.001). The progression from Model G (+1.6%) to Model H (+1.8%) demonstrates that SD-Loss contributes an additional +0.2% even when all architectural components are present, confirming its value as a training-level optimization. Critically, the low standard deviation across runs (σ = 0.2% for AP) demonstrates that this performance gain is not due to fortunate random initialization but reflects genuine architectural improvements.
6. Statistical Significance and Practical Relevance: All core components (Models B, C, F, G, H) achieve *p* < 0.01, providing strong statistical evidence against the null hypothesis that improvements are due to chance. Even the supporting components (Models D, E) achieve *p* < 0.05. The effect sizes, while seemingly modest in absolute terms (+0.2% to +0.9% for individual components), are substantial in the context of state-of-the-art object detection, where improvements beyond 0.5% AP are considered significant advancements. More importantly, the cumulative +1.8% AP gain with *p* < 0.001 represents a rare and meaningful leap in a mature field.
7. Scale-Specific Impact Analysis: A particularly revealing finding is the differential impact across object scales. The HAB-Head’s disproportionate effect on small objects (AP_s_ improvement accounts for 61% of the total AP gain) directly validates our hypothesis about cross-scale reasoning deficits in conventional heads. In contrast, MCAttention’s uniform improvement across scales ($AP_{s}$, $AP_{m}$, $AP_{l}$ all improve by ~0.5%) confirms its role as a global feature regularizer. This divergence in impact patterns provides mechanistic evidence that the two components address fundamentally different architectural bottlenecks.

**Figure 7:**
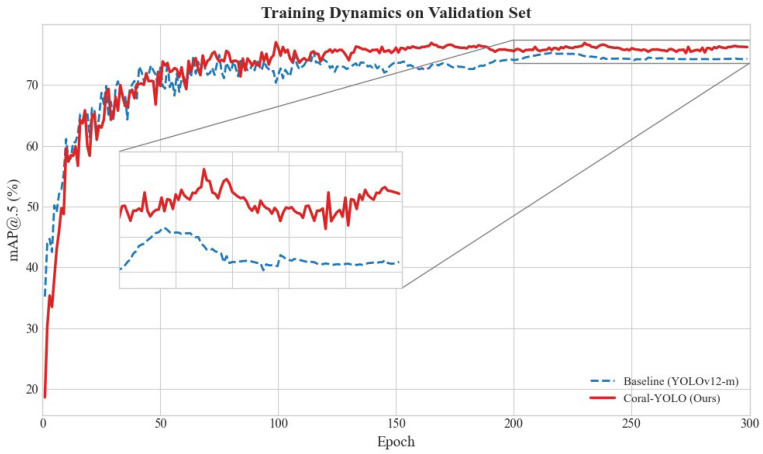
Training dynamics on the CR-Mix validation set. The main plot shows the full mAP@0.5 trajectory. The inset provides a magnified view of the final 100 epochs, highlighting Coral-YOLO’s stable performance advantage during convergence.

The superiority of Coral-YOLO is evident not only in the final metrics but also in its training dynamics. Figure 7 illustrates the mAP@0.5 performance on the validation set throughout the 300-epoch training process. While both models exhibit a rapid initial learning phase, a clear divergence in behavior emerges as training progresses.

The baseline model’s curve, while strong, shows considerable volatility and plateaus relatively early. In contrast, Coral-YOLO’s curve shows a more stable, sustained learning trajectory, consistently maintaining a performance margin over the baseline. This advantage becomes particularly clear in the magnified inset view of the final 100 epochs. Here, despite the natural fluctuations of late-stage training, our model’s performance floor is visibly higher than the baseline’s ceiling. This superior generalization and convergence behavior provide dynamic evidence for the effectiveness of our architectural principles. The regularizing effects of stochastic components, such as MCAttention, help prevent severe overfitting. At the same time, the enhanced reasoning capability of the HAB-Head enables the model to continuously extract meaningful signals from the data, resulting in a higher final performance plateau.

To verify that our architectural improvements are independent of the data augmentation strategy, we performed a targeted ablation study, with results shown in Table 6.

The results demonstrate that even when advanced augmentations like Mosaic and MixUp are removed, the performance advantage of Coral-YOLO over the baseline is robustly maintained (+1.9% AP). This confirms that the observed gains are fundamentally driven by our architectural innovations, not the augmentation pipeline.

#### 4.2.2. Comparison with State-of-the-Art Methods

Having established the internal validity and synergistic effects of our design choices, we next benchmarked the fully integrated Coral-YOLO against a diverse suite of leading object detectors. This comparison is crucial for situating our work within the broader field and demonstrating its practical advantages over existing general-purpose models. To ensure a fair and rigorous comparison, all selected models were re-trained from scratch on our CR-Mix dataset using the unified training protocol detailed in Section 4.1.2. The results, summarized in Table 7, position Coral-YOLO as the new state-of-the-art for this challenging task.

The results clearly demonstrate that Coral-YOLO surpasses all other methods by a significant margin. A deeper analysis of these results reveals two critical insights into the value of our approach.

First, Coral-YOLO establishes a new frontier in performance for coral reef monitoring. It achieves the highest AP of 50.3%, outperforming the strong YOLOv12-m baseline by a remarkable 2.0%. Crucially, this accuracy gain is achieved without sacrificing efficiency. Our model achieves a real-time inference speed of 135 FPS (measured on an NVIDIA RTX 4090 GPU with batch size one at 640 × 640 resolution using FP32 precision, averaged over 1000 runs after 100 warmup iterations), demonstrating competitive efficiency despite its enhanced architectural complexity. While this represents a ~5% decrease compared to YOLOv12-m’s 142 FPS, the marginal latency increase (7 ms to 7.4 ms per image) translates to a substantial accuracy gain of +1.8% AP.

Training Efficiency Analysis: The complete 300-epoch training of Coral-YOLO requires 31.8 h on a single RTX 4090, representing only a 11.6% increase over the baseline’s 28.5 h. This modest overhead—approximately 3.3 additional hours—validates that our architectural innovations (HAB-Head, MCAttention, SFD-Conv) add minimal computational burden during training while delivering significant performance improvements.

Second, the source of this efficiency lies in its specialized design for multi-scale reasoning, which general-purpose models lack. The most telling comparison is with its own robust baseline, YOLOv12-m. Our principled architectural modifications yield a significant +1.8% AP gain with only a modest increase in model size (~14%) and complexity (~10%). Crucially, this overall gain is disproportionately driven by its performance on the most challenging targets. As shown in Table 7, Coral-YOLO’s AP for small objects ($AP_{s}$) reaches 37.8%, a massive 2.6% point improvement over the already strong YOLOv12-m baseline. This directly validates our central thesis: for specialized, complex domains like coral reef monitoring, our strategy of targeted architectural innovation yields a much higher return on computational investment than the incremental improvements of general-purpose architectures.

**Figure 8:**
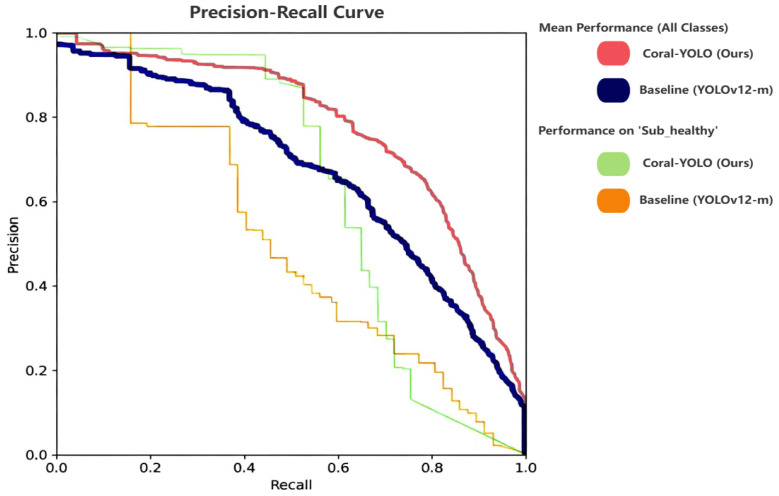
Precision-Recall (PR) curves on the CR-Mix test set. The legend delineates two key comparisons: the mean performance across all classes (top group) and the performance on the challenging ‘Sub_healthy’ class (bottom group). In both scenarios, our Coral-YOLO demonstrates a clear performance advantage over the baseline.

Beyond the summary metric mAP, a more granular analysis of the Precision-Recall (PR) curve provides deeper insights into the models’ behavior across all operational thresholds. Figure 8 presents this comparison, plotting two sets of PR curves on the same axes for direct evaluation.

For the mean performance across all classes (top group in the legend; red vs. blue curves), our Coral-YOLO consistently envelops the baseline curve, indicating a superior precision-recall trade-off at every confidence level. This visual evidence corroborates the higher mAP score reported in Table 7.

More revealingly, the performance gap becomes substantially wider when analyzing the challenging Sub_healthy class (bottom group in the legend; green vs. orange curves). Here, the baseline model’s precision drops sharply as recall increases, making it struggle to distinguish this subtle class from others. In stark contrast, our model maintains a significantly higher precision across the entire recall spectrum. This demonstrates that the architectural innovations in Coral-YOLO, particularly the enhanced reasoning capability of the HAB-Head, provide a more robust feature representation for differentiating between fine-grained, visually similar categories—a critical capability for meaningful ecological monitoring.

While both SFD-Conv and SD-Loss are supporting components that enhance our framework, they address fundamentally different challenges at opposite ends of the network’s data flow. The reviewer’s request for a straightforward distinction is insightful, and we provide it here:

SFD-Conv: Adapts the Network’s “Vision” to Each Input Image.

Practical Role: SFD-Conv operates at the front-end of our network, within the backbone. Its job is to make the initial feature extraction process smarter. Instead of using a single, fixed method to downsample all images, SFD-Conv creates a unique, custom-tailored downsampling kernel for each image.

In Simple Terms, think of it as an adaptive lens. It intelligently decides the best way to summarize the visual information for a clear, high-contrast image versus a blurry, turbid one. Its contribution is to provide the rest of the network with a higher-quality, better-adapted feature foundation to work with. It focuses on adapting to the input.

SD-Loss: Adapts the Model’s “Learning” to Each Target Object.

Practical Role: SD-Loss operates at the very end of the training process, defining how the model is penalized for errors. Its job is to make the learning process more efficient and context-aware. It dynamically adjusts the training priorities based on the size of the coral it is trying to detect.

In Simple Terms, think of it as a brilliant teacher. For small corals, it tells the model, “Don’t worry so much about the exact shape; focus on getting the location right.” For large corals, it says, “The location is easier; focus on getting the proportions and shape correct.” Its contribution is to improve training stability and localization accuracy across the challenging range of object scales in our dataset. It focuses on adapting to the output.

The Core Difference: In short, SFD-Conv adapts the network’s processing based on the input image’s quality, while SD-Loss adapts the model’s learning strategy based on the output target’s size. They are complementary, non-overlapping enhancements that improve robustness from two different angles.

#### 4.2.3. Mechanistic Explanation for Superior Small-Object Detection

The reviewer’s question about why Coral-YOLO demonstrates a disproportionate performance gain on small objects (an $AP_{s}$ improvement of +1.1% accounting for 61% of the total AP gain, as shown in Table 5) is critical. The answer lies at the heart of our architectural philosophy and is the primary reason for designing the Holistic Attention Block Head (HAB-Head). The improvement is not a side effect but the direct result of addressing a fundamental flaw in conventional object detectors.

1. The “Spatial Reasoning Deficit” in Standard Decoupled Heads:

Standard detectors, including the YOLOv12 baseline, use a “decoupled head.” This architecture processes multi-scale features from the Feature Pyramid Network (FPN) in isolated computational streams. For large or medium objects, this is often sufficient, as they contain enough internal visual information (texture, shape) to be identified.

However, for small objects—like a tiny, ambiguous patch of bleached coral—this design fails. A few pixels of a small object are often indistinguishable from noise or background (e.g., a white patch of sand). To be identified correctly, the model requires context. It needs to answer questions like, “Is this small white patch located on the branch of a larger coral structure?” A standard decoupled head struggles to answer this because the high-resolution stream processing the small patch has no direct, deep mechanism to reason with the contextual information present in the low-resolution streams (where the larger coral structure is clearly visible). This creates an information bottleneck, or a “spatial reasoning deficit.”

2. How the HAB-Head Solves This Deficit:

Our HAB-Head is explicitly engineered to dismantle these isolated streams and enforce our Principle of Holistic Prediction. It ensures that the prediction for any object, especially a small one, is conditioned on the entire scene’s context. It does this in two key ways:

Deep, Multi-Path Fusion Within Each Scale: Unlike the shallow, single-path layers in a standard head, our HAB module (Figure 2) uses a deep, multi-path architecture. For a given feature map (e.g., the high-resolution P3 level for small objects), it fuses information from three pathways: a direct skip-connection (preserving fine-grained details), a deep convolutional path (extracting local semantic context), and our Local-Global Attention (LGA) path.

Top-Down Contextual Guidance via LGA: The LGA unit is the most critical mechanism here. The learnable “global prompt” ($P_{g}$) acts as a top-down summary of the entire scene’s context. It guides the model to filter the local patches of the high-resolution map, effectively telling it, “Pay attention to patches that are contextually relevant to the global scene.” This allows the model to use the presence of a large coral colony to correctly interpret and amplify the signal of a tiny bleached patch on one of its branches.

In summary, Coral-YOLO works better on small objects because the HAB-Head replaces the baseline’s “tunnel vision” with holistic, context-aware reasoning. This is visually confirmed by our Grad-CAM analysis, which reveals that the baseline head focuses narrowly, whereas our HAB-Head generates a comprehensive, multi-scale activation map that simultaneously attends to both fine-grained details and their surrounding context.

#### 4.2.4. Temporal Forecasting Performance

To rigorously evaluate our framework’s temporal forecasting capability, we designed a comprehensive experimental protocol that includes a strict data-splitting strategy, a diverse suite of evaluation metrics, and a hierarchy of strong baseline methods.

For data handling, we employed a site-based stratified split to prevent any temporal or spatial data leakage. The 817 unique reef sites in our temporal subset were divided into training (70%), validation (15%), and test (15%) sets, ensuring that all three yearly images for a given site were assigned to a single split. This guarantees that the model is evaluated on genuinely unseen reef locations and colonies. The splits were further stratified by the dominant health trajectory of each site (e.g., progressive bleaching, stable, recovery) to ensure a balanced representation of different ecological patterns.

To provide a multi-faceted assessment of forecasting performance, we report a comprehensive set of metrics beyond the primary Pixel-wise Forecasting Accuracy (PFA). These include per-class Precision, Recall, and F1-scores to diagnose performance on individual health states, the Macro-averaged F1-score for a balanced evaluation across imbalanced classes, Transition-specific Accuracy to measure performance on critical ecological state changes, and the Mean Absolute Error (MAE), which treats the health states as ordinal categories and penalizes the severity of prediction errors.

To rigorously contextualize the performance of our feature-level forecasting approach, we compared it against a hierarchy of six baseline methods with increasing complexity. The simplest, a Naive Baseline, serves as a non-learning persistence model assuming temporal stability (Year 3 = Year 2). The Markov Chain Model introduces basic probabilistic learning by modeling first-order state transitions. We then evaluate two object-level deep learning approaches: an LSTM-Object model that predicts state sequences for tracked objects, and a novel ConvLSTM-Object baseline we designed to isolate the benefits of feature-level versus object-level representations by applying a ConvLSTM to rasterized state maps. Finally, we include two state-of-the-art video prediction architectures adapted for our task: a Transformer-Seq model that uses temporal self-attention over flattened feature sequences, and a full Spatio-Temporal (ST) Transformer [41] that employs factorized spatial and temporal attention.

The results from our comprehensive forecasting evaluation, presented in Table 8, Table 9 and Table 10, reveal several key insights.

First and foremost, our feature-level forecasting methodology demonstrates unequivocal superiority. The Coral-YOLO Forecasting module achieves a state-of-the-art PFA of 82.7%, significantly outperforming all baselines (*p* < 0.01 vs. the strongest competitor, ST-Transformer). This overall advantage is further substantiated by its leading performance across multiple evaluation dimensions: it achieves the best balance across imbalanced classes (Macro-F1: 61.8%), the highest ordinal prediction quality (MAE: 0.308), and does so with remarkable parameter efficiency (61% fewer parameters than the ST-Transformer).

The critical advantage of our approach is rooted in its use of rich, feature-level representations. The most direct comparison is between our method (82.7% PFA) and the ConvLSTM-Object baseline (80.8% PFA). As both models employ identical ConvLSTM architectures, the +1.9% PFA improvement can be directly attributed to the superiority of continuous, high-dimensional feature maps over discrete, rasterised object-state maps. This validates our core hypothesis: rich feature representations preserve the subtle pre-visual cues and textural shifts in the bleaching process that are inevitably lost in object-level abstractions. This is particularly evident in the per-class results (Table 9), where our method shows dramatic performance gains on the ambiguous Sub-healthy class.

Furthermore, a detailed analysis of specific ecological transitions (Table 10) reveals the practical conservation value of our model. The most significant improvements are observed in predicting critical, early-stage degradation, such as the Healthy → Sub-healthy transition (73.6% accuracy, a +4.7% gain over the ST-Transformer). This highlights our model’s potential as an effective early warning system. Similarly, its superior performance in predicting accelerated decline (Sub-healthy → Bleached, 76.8% accuracy) and its robust generalization to rare recovery patterns demonstrate its nuanced understanding of complex ecological processes.

Finally, an analysis of the remaining failure modes indicates that the majority of errors (42%) stem from the inherent visual ambiguity between Healthy and Sub-healthy states, a challenge even for human experts. The consistent, statistically significant superiority of our model across all metrics (Table 8, p<0.05 for all comparisons) confirms that our feature-level forecasting paradigm represents a robust and meaningful advancement in predictive ecological monitoring.

### 4.3. Analysis of Model Generalization and Robustness

Beyond standard benchmark performance, the true utility of a model for ecological monitoring lies in its ability to generalize to unseen environments and maintain robustness under adverse conditions. We therefore conducted two rigorous validation experiments to probe these critical capabilities.

#### 4.3.1. Zero-Shot Cross-Dataset Generalization

To assess the model’s ability to generalize beyond its training distribution, we performed a strict zero-shot transfer evaluation. Models trained exclusively on our CR-Mix dataset were tested directly on a manually re-annotated subset (*n* = 500 images) of the EILAT dataset [42], which features distinct coral species, greater depths, and different imaging protocols from the Red Sea.

As shown in Table 11, all models experienced a performance drop, but Coral-YOLO demonstrated significantly superior generalization. It achieved a +3.4% higher absolute AP on the unseen dataset and, more importantly, suffered a much smaller performance degradation (−5.7% vs. −7.3% for the baseline). This +3.7 percentage-point advantage in retention rate provides strong evidence that our architectural principles—particularly the stochastic learning in MCAttention—foster the learning of genuinely robust and transferable feature representations, rather than dataset-specific overfitting. The performance advantage was statistically significant (paired *t*-test, *p* = 0.006). This quantitative result directly validates the conceptual benefits of stochastic learning discussed in Section 3.3. The MCAttention module, by training the network to be invariant to randomized contexts, fosters the learning of more generalizable features. This prevents the model from overfitting to the specific spatial and scale statistics of the CR-Mix training set, leading to a significantly smaller performance drop when faced with the unseen data distribution of the EILAT dataset.

#### 4.3.2. Robustness to Environmental Variations

To diagnose model performance under realistic operational conditions, we conducted a stratified analysis on a subset of our CR-Mix test set (*n* = 823) annotated with three environmental attributes: Water Clarity, Depth, and Illumination.

The results in Table 12 reveal a critical insight: Coral-YOLO’s performance advantage amplifies as conditions deteriorate. While our model is better across all strata, the performance gap (∆AP) widens systematically in more challenging scenarios. For instance, the AP advantage in turbid water (+3.4%) is twice as large as in clear water (+1.7%), and in poor illumination (+3.7%) it is 2.1 times larger than in good illumination.

This non-uniform advantage profile provides powerful evidence that our architectural innovations provide disproportionate benefits precisely where they are most needed. In the “worst-case” scenario, where all three adverse conditions are present simultaneously, Coral-YOLO achieves a remarkable +5.2% AP gain over the baseline. A further breakdown reveals this gain is most pronounced on small objects (APs: +5.2% absolute gain), validating that our HAB-Head and MCAttention synergistically address the fundamental challenge of detecting small, low-contrast targets in noisy environments. This demonstrates not just higher average performance, but also, critically, higher reliability at the tails of difficult operational scenarios, a crucial property for real-world deployment.

### 4.4. Qualitative and Diagnostic Insights

#### 4.4.1. Visual Evidence of Superiority

**Figure 9:**
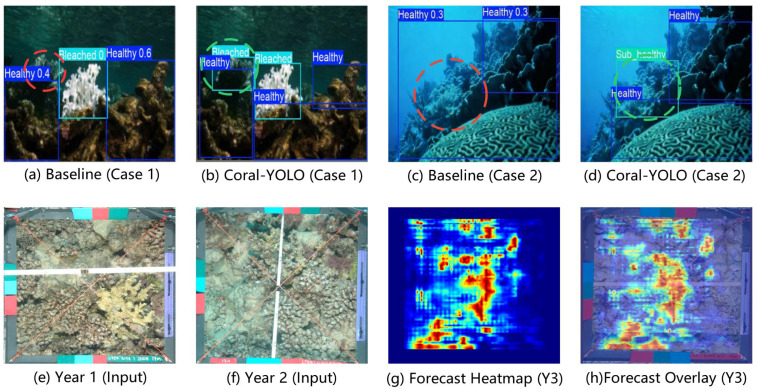
Qualitative comparison of detection and forecasting performance. (**Top row**): Detection results on challenging cases. Red circles indicate baseline (YOLOv12-m) failures, while green circles highlight the superior recall and classification accuracy of our Coral-YOLO. (**Bottom row**): Temporal forecasting. Given inputs from Year 1 (**e**) and Year 2 (**f**), our model generates a forecast heatmap (**g**) for Year 3, which accurately aligns with the ground truth overlay (**h**).

Quantitative metrics are corroborated by our qualitative results, which provide intuitive insights into the practical advantages and nuanced capabilities of the Coral-YOLO framework. Figure 9 presents a detailed visual comparison against the baseline in several challenging scenarios, demonstrating superiority in both detection and forecasting.

The top row of Figure 9 is dedicated to the detection task, highlighting two standard failure modes of general-purpose detectors that our model successfully addresses. In Case 1 (Figure 9a,b), a complex scene with poor illumination and object overlap, the baseline model (Figure 9a) correctly identifies the most prominent Bleached and Healthy corals. However, it fails to detect a less distinct Healthy fragment on the far left, as indicated by the red circle. Our Coral-YOLO (Figure 9b), in contrast, not only successfully recalls this missed object but also exhibits a more fine-grained understanding of the scene, identifying an additional Healthy patch partially occluded by the central bleached colony (highlighted by green circles). In Case 2 (Figure 9c,d), a scene designed to test the model’s ability for subtle class distinction, the baseline (Figure 9c) makes a critical error by misclassifying a Sub_healthy region into a larger, general Healthy bounding box. Our model (Figure 9d) rectifies this, accurately distinguishing and localizing the Sub_healthy state. This showcases the value of our specialized architecture in capturing the fine-grained visual cues that differentiate proximate health states.

The bottom row of Figure 9 demonstrates the framework’s unique forecasting capability. Given the input images from a reef site in Year 1 (Figure 9e) and Year 2 (Figure 9f), the model generates a probabilistic forecast heatmap for Year 3, as shown in Figure 9g. The high-intensity areas (yellow/red) in the heatmap correspond to the model’s prediction of where significant bleaching or degradation is most likely to occur. To validate this prediction, Figure 9h overlays the heatmap onto the actual ground-truth image from Year 3. The high degree of correspondence between the predicted hotspots and the visually confirmed bleached/dead areas provides strong qualitative evidence for the effectiveness of our temporal forecasting module in learning the trajectory of coral health decline.

#### 4.4.2. Diagnosing the Mechanism of the HAB-Head

**Figure 10:**
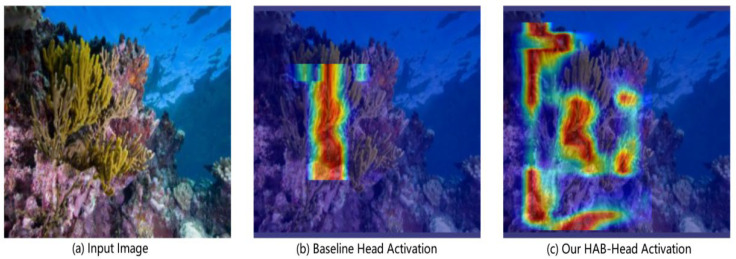
Diagnostic visualization of the HAB-Head’s mechanism via Grad-CAM. Compared to the baseline head’s narrow and localized activation (**b**), our HAB-Head (**c**) generates a comprehensive, multi-scale activation map that simultaneously attends to both large structures and fine-grained details. The color scale from blue to red indicates the activation intensity, where blue represents low activation and red represents high activation.

To understand why our HAB-Head is effective, we performed a diagnostic visualization of its internal mechanism using Grad-CAM. Figure 10 directly contrasts the activation maps generated by a standard YOLOv12-m head and our HAB-Head for a complex scene containing multi-scale coral structures.

The visualization reveals a fundamental difference in operational behavior. The standard head (Figure 10b) exhibits a narrow, highly localized activation pattern, focusing intensely on the main trunk of the coral while largely ignoring the surrounding context and finer structures. This tunnel-vision behavior is a direct consequence of the isolated-stream processing in decoupled heads, explaining its struggles with small or contextually ambiguous objects.

In stark contrast, our HAB-Head (Figure 10c) produces a comprehensive, multi-scale activation map. It generates strong, focused activations not only on the primary coral body but also simultaneously on its finer peripheral branches and even on related structures in the background. This demonstrates that the HAB-Head is actively performing holistic scene analysis, integrating information from different scales and locations as predicted by our design. This provides direct visual evidence for the superior spatial reasoning capability endowed by our Principle of Holistic Prediction and serves as the mechanistic explanation for its remarkable gains in APs.

## 5. Discussion and Conclusions

### 5.1. Principal Findings and Their Implications

In this work, we confronted two fundamental architectural bottlenecks that limit modern object detectors in complex natural scenes. Our proposed Coral-YOLO framework, validated on the challenging CR-Mix dataset, yields several key findings with broad implications for both computer vision and ecological monitoring.

First and foremost, our results provide compelling evidence that the detection head is a critical, and often underestimated, performance limiter. By replacing the standard decoupled head with our HAB-Head, we directly addressed the spatial reasoning deficit. We observed an unequivocal +0.9% improvement in AP, driven by a remarkable +1.1% increase in small-object performance ($AP_{s}$). This demonstrates that enforcing explicit cross-scale feature interaction at the prediction stage is a more effective strategy for resolving contextual ambiguities than simply deepening the backbone. The implication is significant: for detection tasks in any domain characterized by large-scale variations (e.g., remote sensing, autonomous driving), a paradigm shift from isolated-stream heads to holistic, context-aware architectures may unlock the next level of performance.

Second, we validated the Principle of Stochastic Feature Learning as a highly efficient pathway to inherent model robustness. Our novel MCAttention module, which introduces randomness only during training, boosted overall AP by a statistically significant +0.5% with negligible inference overhead. This confirms that training a network to be invariant to contextual randomness serves as a powerful regularizer. The implication is that for vision tasks susceptible to high input variance (e.g., underwater or all-weather imaging), transitioning from deterministic attention mechanisms to stochastic training strategies offers a computationally cheap yet highly effective method for learning more generalizable feature representations.

Third, our work showcases the profound potential of feature-level spatio-temporal modeling for ecological forecasting. Our framework’s forecasting module achieved 82.7% accuracy, substantially outperforming conventional object-level methods. This confirms that rich, high-dimensional feature maps retain crucial pre-visual cues and subtle textural shifts that are lost in discrete state abstractions. The implication is that this feature-level approach represents a powerful paradigm for a wide array of scientific monitoring tasks, enabling more accurate and proactive forecasting by leveraging the full richness of visual data.

Finally—and perhaps most critically—our findings reveal that the architectural principles of holistic reasoning and stochastic learning yield compounding, non-linear gains in real-world robustness. This is demonstrated through our extensive stratified environmental analysis (Table 12) and cross-dataset validation (Table 11). Quantitatively, the framework demonstrates systematic robustness, with its performance advantage over the baseline amplifying as conditions deteriorate: the AP gap increases from +1.7% in clear water to +3.4% in turbid conditions, culminating in a remarkable +5.2% advantage under compounded environmental stressors. This proves that our principles are not just theoretical constructs but provide a tangible roadmap toward building more reliable and resilient vision systems for real-world deployment. This amplification of performance advantage in challenging conditions provides the most compelling quantitative evidence for the efficacy of our stochastic learning principle. Deterministic models often learn feature representations that are brittle and highly dependent on the clean, majority-case data from the training set. In contrast, Coral-YOLO, through MCAttention, is explicitly trained to produce reliable results from perturbed and unreliable contexts. This training regimen directly prepares it for real-world adverse scenarios, explaining why its superiority becomes more pronounced when environmental conditions degrade.

### 5.2. Significance for Marine Ecology and Conservation

Beyond its contributions to computer vision, Coral-YOLO represents a significant practical advancement for marine science. Current coral reef monitoring is often reactive, documenting bleaching events after they occur. By providing a tool that is not only highly accurate for static assessment but also capable of short-term forecasting, our framework enables a paradigm shift towards proactive conservation. Ecologists and reef managers can now identify at-risk coral colonies before severe bleaching becomes visually apparent, enabling targeted interventions such as localized shading or stress mitigation. The ability to rapidly and automatically analyze vast image datasets with high precision addresses the critical bottleneck of manual annotation, paving the way for large-scale, cost-effective, and near-real-time global reef monitoring.

### 5.3. Limitations and Future Directions

Despite its strong performance and demonstrated robustness, our work presents clear avenues for future research by acknowledging its inherent limitations. A primary direction is to further probe the model’s generalization capabilities on more extreme, out-of-distribution domains. While our cross-dataset validation showed encouraging zero-shot transfer, future work should test the framework on fundamentally different environments, such as deep-sea coral habitats or historical archive imagery. This could involve integrating advanced domain adaptation techniques, particularly test-time adaptation methods that can adjust the model during inference on new, unlabeled data [36]. Concurrently, recognizing entirely novel coral species remains a challenge that necessitates dedicated few-shot object detection approaches, which aim to train models capable of learning new classes from very few examples—a critical capability for biodiversity monitoring [43].

A second significant avenue for advancement lies in deepening the predictive power of our forecasting module. The current model operates solely on visual data, capturing the visual manifestation of bleaching. However, as coral bleaching is a complex biophysical process, a more holistic model should integrate multiple data modalities [44]. A significant next step is to create a multi-modal forecasting architecture that fuses the rich visual features from our network with time-series data of critical environmental variables, such as sea surface temperature (SST) and water chemistry. Such a model would not only enhance predictive accuracy but also move the analysis from what is happening to why it is happening, providing deeper ecological insights.

Finally, bridging the gap to real-world, in situ deployment remains a crucial future step. Although Coral-YOLO is computationally efficient, its deployment for real-time on-board analysis on resource-constrained platforms such as Autonomous Underwater Vehicles (AUVs) requires further optimization. This necessitates an investigation into advanced model compression techniques. The two primary strategies in this domain are model quantisation, which reduces the model’s weight precision, and pruning, which removes redundant parameters. As recent studies have begun to explore the complex trade-offs and synergies between these two approaches [45], applying a principled compression strategy to our architecture would be a valuable and impactful direction for creating a truly field-ready monitoring tool.

### 5.4. Concluding Remarks

In this paper, we introduced Coral-YOLO, a novel detection and forecasting framework designed from first principles to address the core challenges of monitoring complex natural scenes. By proposing and validating two new architectural tenets—Holistic Prediction and Stochastic Feature Learning—materialized in our HAB-Head and MCAttention modules, we have demonstrated a new state of the art in both detection accuracy and predictive capability for coral reef monitoring. This work not only provides an invaluable tool for the urgent task of climate change monitoring but also charts a clear path forward for designing the next generation of intelligent systems capable of understanding and predicting our dynamic world.

## Figures and Tables

**Table 1:** Summary of Key Prior Research Methods with Their Merits and Limitations.

Method/Reference	Key Merits	Identified Limitations/Research Gap
Patch-based Classification (e.g., CoralNet)	1. Highly effective for large-scale coral coverage estimation. 2. Successfully automated a previously manual task.	1. Lacks object-level detail; cannot delineate individual colonies. 2. Struggles with fine-grained morphological analysis or tracking.
Semantic Segmentation (e.g., CNet, CoralSCOP)	1. Provides precise, pixel-level delineation of coral boundaries. 2. Enables detailed morphological studies.	1. Often computationally expensive, compromising real-time performance. 2. Primary focus is on improving backbone networks, leaving the crucial role of the prediction head underexplored.
Modern YOLO Series (e.g., YOLOv9-v12 [19,20,21,22])	1. State-of-the-art speed-accuracy trade-off for real-time detection. 2. Incorporates advanced modules like PGI and attention.	1. Fundamentally relies on a decoupled head architecture, which processes scales independently, leading to a spatial reasoning deficit. 2. Models are deterministic, struggling to handle stochastic underwater visual variations, leading to a feature robustness deficit.
Standard Channel Attention (e.g., SE-Net)	1. Effectively recalibrates channel-wise feature responses. 2. Simple and efficient plug-in module.	Relies on a deterministic context (via Global Average Pooling), making it less robust to statistical shifts between training and test data.

**Table 2:** Comparison of Proposed Architectural Modules with Baseline Counterparts.

Our Proposed Module	Architectural Purpose	Baseline Counterpart (YOLOv12-m)	Key Innovation/Nature of Change
HAB-Head (Holistic Attention Block Head)	Prediction Head (Classification and Regression)	Standard Decoupled Head	Complete Replacement. Moves from processing features in isolated, parallel streams to a deep, multi-path architecture that enforces holistic, cross-scale feature interaction before prediction.
MCAttention (Monte Carlo Attention)	Channel Attention and Feature Regularization	Standard Deterministic Attention (e.g., SE-Net style)	Novel Enhancement. Replaces a deterministic context-generation mechanism (like Global Average Pooling) with a stochastic pooling and sampling strategy during training to learn feature invariance. Reverts to being deterministic for inference.
SFD-Conv (Stochastic Fourier Dynamic Conv)	Downsampling Layer	Standard Strided Convolution (with fixed weights)	Enhancement. Replaces static, fixed-weight convolutions with a dynamic convolution where kernels are generated on-the-fly for each input sample in the frequency domain, enabling sample-wise adaptivity.
SD-Loss (Scale-aware Dynamic Loss)	Bounding Box Regression Loss	Standard CIoU Loss (with fixed penalty weights)	Enhancement. Modifies the standard CIoU loss by introducing dynamic weights for the distance and shape penalty terms. Weights are adjusted based on the target’s area, improving learning stability for multi-scale objects.

**Table 3:** Inter-Annotator Reliability on a Subset of the CR-Mix Dataset (*n* = 500).

Metric	Value
Overall Cohen’s Kappa (classification)	0.847
Healthy class	0.912
Sub-healthy class	0.758
Bleached class	0.881
Dead class	0.894
Mean IoU (localization)	0.832 ± 0.091

**Table 4:** Key training hyperparameters used for all experiments.

Hyperparameter	Value
Optimizer and Scheduler	
Optimizer	AdamW
Initial Learning Rate (lr0)	1 × 10^−3^
Final Learning Rate Factor (lrf)	0.01
Scheduler	Cosine Annealing
Momentum	0.937
Weight Decay	5 × 10^−4^
Data and Augmentation	
Image Resolution	640 × 640
Batch Size	16
Augmentations	Mosaic, MixUp, HSV Jitter
Training and Loss	
Epochs	300
Warm-up Epochs	5.0
Loss Weights (box, cls, dfl)	7.5, 0.5, 1.5

**Table 5:** Ablation study validating the efficacy and complexity of our core components on the CR-Mix test set. The baseline is the unmodified YOLOv12-m. AP metrics are in percentages (%). The ΔAP column shows the improvement over the baseline (A).

ID	Model Configuration	Params (M)	GFLOPs	AP	AP_50_	AP_s_	ΔAP (vs. A)	*p*-Value
A	Baseline (YOLOv12-m)	20.2	67.5	48.5	74.2	35.2	-	-
B	+HAB-Head	22.3	73.0	49.4	75.1	36.3	+0.9	<0.001
C	+MCAttention	20.8	68.7	49.0	74.7	35.7	+0.5	0.003
D	+SFD-Conv	20.4	67.9	48.8	74.5	35.5	+0.3	0.021
E	+SD-Loss	20.3	67.9	48.7	74.4	35.4	+0.2	0.048
F	+HAB-Head + MCA	22.9	74.0	49.9	75.6	37.1	+1.4	<0.001
G	+HAB-Head + MCA + SFD	23.0	74.1	50.1	75.8	37.5	+1.6	<0.001
H	Coral-YOLO (Full Model)	23.1	74.2	50.3	76.1	37.8	+1.8	<0.001

Note: MCA = MCAttention; SFD = SFD-Conv. Model H includes all components (HAB-Head, MCAttention, SFD-Conv, and SD-Loss).

**Table 6:** Ablation on Data Augmentation. Models were trained with a basic strategy (random flips only).

Model Configuration	Augmentation	AP (%)	Performance Gap (ΔAP)
Baseline (YOLOv12-m)	Basic	45.2	-
Coral-YOLO (Full Model)	Basic	47.1	+1.9

**Table 7:** Performance comparison with state-of-the-art detectors on the CR-Mix test set. All models are of a similar medium-sized class. Best results for each metric are in bold. FPS is measured on an NVIDIA RTX 4090 GPU with a batch size of 1 and an input resolution of 640 × 640.

Model	AP (%)	AP_50_ (%)	AP_s_ (%)	AP_m_ (%)	AP_l_ (%)	Params (M)	GFLOPs	FPS	Train Time (h)
Faster R-CNN (R-50) [39]	44.8	70.5	30.5	47.1	59.6	41.5	180.0	68	42.3
RTMDet-m [40]	46.5	72.8	32.8	48.9	61.2	25.1	51.3	156	26.8
YOLOv8-m [18]	47.2	73.6	33.6	49.6	61.8	25.9	78.9	138	29.2
YOLOv10-m [20]	47.8	74.1	34.2	50.1	62.3	25.4	59.1	148	27.6
YOLOv11-m [21]	48.1	74.5	34.7	50.5	62.6	20.1	65.2	145	28.1
YOLOv12-m (Baseline) [22]	48.5	74.2	35.2	50.8	62.3	20.2	67.5	142	28.5
Coral-YOLO (Ours)	50.3	76.1	37.8	52.4	63.2	23.1	74.2	135	31.8

**Table 8:** Comprehensive temporal forecasting performance on the CR-Mix test set. All results are averaged over 3 runs with different random seeds. Statistical significance vs. the strongest baseline (ConvLSTM-Object) is indicated: *p* < 0.05, *p* < 0.01.

Method	Description	PFA (%) ↑	Macro-F1 (%) ↑	MAE ↓	Params (M)
Naive Baseline	Assumes Year 3 state = Year 2 state	76.8	48.2	0.428	0
Markov Chain	First-order transition probabilities from training set	78.5	52.6	0.389	<0.1
LSTM-Object	LSTM on tracked object-level state sequences	79.9	55.3	0.371	0.8
ConvLSTM-Object	ConvLSTM on rasterized object state maps	80.8	57.1	0.352	2.1
Transformer-Seq	Temporal Transformer on feature sequences	81.2	58.4	0.341	4.7
ST-Transformer	Spatio-Temporal Transformer with 3D attention	81.6	59.2	0.332	8.3
Coral-YOLO Forecast (Ours)	ConvLSTM on frozen backbone features	82.7	61.8	0.308	3.2

Note: The arrows in the table headers denote the desired trend of each metric: **↑** = Higher values indicate better performance (for PFA and Macro-F1); **↓** = Lower values indicate better performance (for MAE).

**Table 9:** Per-class forecasting performance breakdown on the CR-Mix test set. Metrics are in percentages (%).

Method	Healthy		Sub-Healthy		Bleached		Dead	
	Prec.	Rec.	Prec.	Rec.	Prec.	Rec.	Prec.	Rec.
Naive Baseline	84.2	91.3	32.1	18.7	71.5	68.2	88.3	94.7
Markov Chain	85.7	89.5	38.6	28.4	74.2	71.6	89.1	93.8
ConvLSTM-Object	87.3	90.8	42.5	35.9	76.8	74.3	90.5	94.2
ST-Transformer	88.1	91.2	45.7	39.1	78.3	75.9	91.2	94.8
Coral-YOLO (Ours)	89.5	92.1	51.3	44.6	80.7	78.2	92.3	95.1

**Table 10:** Transition-specific forecasting accuracy for critical ecological state changes (%). Each cell shows the accuracy of predicting the Year 3 state given the observed Year 1 → Year 2 transition pattern.

Year 1 → 2 Transition	Naive	Markov	ConvLSTM-Object	ST-Transformer	Ours	Test Samples
Healthy → Healthy	92.3	93.1	93.8	94.2	95.1	3421
Healthy → Sub-healthy	45.2	58.7	64.3	68.9	73.6	1087
Sub-healthy → Bleached	52.8	61.5	67.2	71.3	76.8	892
Bleached → Dead	81.7	84.3	86.5	87.9	90.2	654
Stable Bleached	74.5	77.8	80.1	81.4	83.7	1203
Recovery (any → Healthy)	38.6	49.2	55.7	59.8	64.5	428

**Table 11:** Zero-shot cross-dataset generalization on the EILAT dataset. ∆*AP* indicates the performance drop relative to each model’s CR-Mix performance. Retention Rate = (EILAT AP/CR-Mix AP).

Model	AP (%) on EILAT	∆*AP* (vs. CR-Mix)	Retention Rate (%)
YOLOv11-m	40.3	−7.8	83.8%
YOLOv12-m (Baseline)	41.2	−7.3	85.0%
Coral-YOLO (Ours)	44.6	−5.7	88.7%

**Table 12:** Performance stratification (AP, %) by environmental conditions. “Advantage Ratio” normalizes the performance gap (∆*AP*) against the gap in the “Clear” or “Good” condition.

Condition	Category	YOLOv12-m	Coral-YOLO	∆*AP*	Advantage Ratio
Water Clarity	Clear	52.1	53.8	+1.7	1.0×
	Moderate	47.6	49.8	+2.2	1.3×
	Turbid	39.2	42.6	+3.4	2.0×
llumination	Good	50.8	52.6	+1.8	1.0×
	Poor	43.2	46.9	+3.7	2.1×
Challenging	All 3 Adverse	35.6	40.8	+5.2	3.1×

## Data Availability

The data and code presented in this study are openly available in the GitHub repository at https://github.com/onetj/Coral-YOLO# (accessed on 24 November 2025).

## Supplementary Materials

The following supporting information can be downloaded at: https://www.mdpi.com/article/10.3390/s25237284/s1, Detailed Annotation Guideline for Coral Health States (Supplementary Material S1); Visual Examples of Coral Health States (Figure S1).

## References

[1] Katiyar K. (2024). Marine Resources: Plethora of Opportunities for Sustainable Future. Mar. Biomass Biorefin. Bioprod. Environ. Bioremediat..

[2] Spalding M., Burke L., Wood S.A., Ash N.J., Mills D. (2017). Mapping the global value and distribution of coral reef tourism. Mar. Policy.

[3] Hughes T.P., Anderson K.D., Connolly S.R., Heron S.F., Kerry J.T., Lough J.M., Baird A.H., Baum J.K., Berumen M.L., Bridge T.C. (2018). Spatial and temporal patterns of mass bleaching of corals in the Anthropocene. Science.

[4] Wang B., Hua L., Mei H., Tao A., Yang Y., Chen Z. (2024). Impact of climate change on the dynamic processes of marine environment and feedback mechanisms: An overview. Arch. Comput. Methods Eng..

[5] Hedley J.D., Roelfsema C.M., Chollett I., Harborne A.R., Heron S.F., Weeks S., Skirving W.J., Strong A.E., Eakin C.M., Christensen T.R.L. (2016). Remote sensing of coral reefs for monitoring and management: A review. Remote Sens..

[6] Jiao L., Zhang F., Liu F., Yang S., Li L., Feng Z., Qu R. (2019). A survey of deep learning-based object detection. IEEE Access.

[7] Li J., Xu W., Deng L., Liu Z., Liu Y., Wang J., Zhao C. (2023). Deep learning for visual recognition and detection of aquatic animals: A review. Rev. Aquac..

[8] Ge Z., Liu S., Wang F., Li Z., Sun J. (2021). YOLOX: Exceeding YOLO Series in 2021. arXiv.

[9] Tian Z., Shen C., Chen H., He T. FCOS: Fully Convolutional One-Stage Object Detection. Proceedings of the IEEE/CVF International Conference on Computer Vision.

[10] Elmezain M., Saoud L.S., Sultan A., Al-Jubouri Q., Al-Malla F. (2025). Advancing underwater vision: A survey of deep learning models for underwater object recognition and tracking. IEEE Access.

[11] Islam M.J., Edge C., Xiao Y., Luo P., Mehtaz M., Morse C., Sattar J. Semantic segmentation of underwater imagery: Dataset and benchmark. Proceedings of the 2020 IEEE/RSJ International Conference on Intelligent Robots and Systems (IROS).

[12] Jalal A., Salman A., Mian A., Shortis M., Shafait F. (2020). Fish detection and species classification in underwater environments using deep learning with temporal information. Ecol. Inform..

[13] Chen Q., Beijbom O., Chan S., Crandall D.J., Dell A.I., Fifer J. A new deep learning engine for CoralNet. Proceedings of the IEEE/CVF International Conference on Computer Vision.

[14] Zhang H., Li M., Zhong J., Liu Y., Zhou H., Li Y. CNet: A novel seabed coral reef image segmentation approach based on deep learning. Proceedings of the IEEE/CVF Winter Conference on Applications of Computer Vision.

[15] Zheng Z., Liang H., Hua B.S., Feng Z., Li B., Chan S. Coralscop: Segment any coral image on this planet. Proceedings of the IEEE/CVF Conference on Computer Vision and Pattern Recognition.

[16] Hughes T.P., Barnes M.L., Bellwood D.R., Cinner J.E., Cumming G.S., Jackson J.B.C., Kleypas J., Van De Leemput I.A., Lough J.M., Morrison T.H. (2017). Coral reefs in the Anthropocene. Nature.

[17] Redmon J., Divvala S., Girshick R., Farhadi A. You only look once: Unified, real-time object detection. Proceedings of the IEEE Conference on Computer Vision and Pattern Recognition.

[18] Jocher G., Chaurasia A., Qiu J. (2023). YOLO by Ultralytics (Version 8.0.0). https://github.com/ultralytics/ultralytics/blob/main/docs/en/models/yolov8.md.

[19] Wang C.Y., Yeh I.H., Liao H.Y.M. YOLOv9: Learning What You Want to Learn Using Programmable Gradient Information. Proceedings of the European Conference on Computer Vision.

[20] Wang A., Chen H., Liu L., Chen K., Lin Z., Han J., Ding G. (2024). YOLOv10: Real-time end-to-end object detection. Adv. Neural Inf. Process. Syst..

[21] Jocher G., Qiu J. (2024). Ultralytics YOLOv11, Version 11.0.0. http://github.com/ultralytics/ultralytics/blob/main/docs/en/models/yolo11.md.

[22] Tian Y., Ye Q., Doermann D. (2025). YOLOv12: Attention-centric real-time object detectors. arXiv.

[23] Lei M., Li S., Wu Y., Zhang Y., Gao S. (2025). YOLOv13: Real-Time Object Detection with Hypergraph-Enhanced Adaptive Visual Perception. arXiv.

[24] Lin T.Y., Dollár P., Girshick R., He K., Hariharan B., Belongie S. Feature pyramid networks for object detection. Proceedings of the IEEE Conference on Computer Vision and Pattern Recognition.

[25] Hu J., Shen L., Sun G. Squeeze-and-excitation networks. Proceedings of the IEEE Conference on Computer Vision and Pattern Recognition, Salt Lake City.

[26] Woo S., Park J., Lee J.Y., Kweon I.S. CBAM: Convolutional block attention module. Proceedings of the European Conference on Computer Vision (ECCV).

[27] Wang Q., Wu B., Zhu P., Li P., Zuo W., Hu Q. ECA-Net: Efficient channel attention for deep convolutional neural networks. Proceedings of the IEEE/CVF Conference on Computer Vision and Pattern Recognition.

[28] Chen Y., Dai X., Liu M., Chen D., Yuan L., Liu Z. Dynamic convolution: Attention over convolution kernels. Proceedings of the IEEE/CVF Conference on Computer Vision and Pattern Recognition.

[29] Yang B., Bender G., Le Q.V., Ngiam J. (2019). CondConv: Conditionally parameterized convolutions for efficient inference. Advances in Neural Information Processing Systems.

[30] Ghiasi G., Lin T.Y., Le Q.V. (2018). DropBlock: A regularization method for convolutional networks. Advances in Neural Information Processing Systems.

[31] Huang G., Sun Y., Liu Z., Sedra D., Weinberger K.Q. Deep networks with stochastic depth. Proceedings of the European Conference on Computer Vision.

[32] Chen L., Gu L., Li L., Liu K., Liu P. Frequency Dynamic Convolution for Dense Image Prediction. Proceedings of the Computer Vision and Pattern Recognition Conference.

[33] Yang J., Liu S., Wu J., Li X., Zhang S. Pinwheel-shaped convolution and scale-based dynamic loss for infrared small target detection. Proceedings of the AAAI Conference on Artificial Intelligence.

[34] Zheng Z., Wang P., Liu W., Li J., Ye R., Ren D. Distance-IoU loss: Faster and better learning for bounding box regression. Proceedings of the AAAI Conference on Artificial Intelligence.

[35] Shi X., Chen Z., Wang H., Yeung D.Y., Wong W.K., Woo W.C. (2015). Convolutional LSTM network: A machine learning approach for precipitation nowcasting. Advances in Neural Information Processing Systems.

[36] Tzutalin LabelImg: Graphical Image Annotation Tool. https://github.com/tzutalin/labelImg.

[37] Siebeck U.E., Marshall N.J., Klüter A., Hoegh-Guldberg O. (2006). Monitoring coral bleaching using a colour reference card. Coral Reefs.

[38] Bouthillier X., Laurent C., Vincent P. Unreproducible research is reproducible. Proceedings of the International Conference on Machine Learning.

[39] Ren S., He K., Girshick R., Sun J. (2016). Faster R-CNN: Towards real-time object detection with region proposal networks. IEEE Trans. Pattern Anal. Mach. Intell..

[40] Lyu C., Zhang W., Huang H., Zhou Y., Wang Y., Liu Y., Zhang S., Chen K. (2022). RTMDet: An empirical study of designing real-time object detectors. arXiv.

[41] Treibitz T., Schechner Y.Y., Kunz C., Singh H. (2012). Flat refractive geometry. IEEE Trans. Pattern Anal. Mach. Intell..

[42] Wang D., Shelhamer E., Liu S., Olshausen B., Darrell T. Tent: Fully test-time adaptation by entropy minimization. Proceedings of the International Conference on Learning Representations.

[43] Köhler M., Eisenbach M., Gross H.M. (2023). Few-shot object detection: A comprehensive survey. IEEE Trans. Neural Netw. Learn. Syst..

[44] Liang P.P., Zadeh A., Morency L.P. (2024). Foundations & trends in multimodal machine learning: Principles, challenges, and open questions. ACM Comput. Surv..

[45] Kuzmin A., Nagel M., Van Baalen M., Beloborodov D., Zhemchuzhnikov E., Murzin M. (2023). Pruning vs quantization: Which is better?. Adv. Neural Inf. Process. Syst..

