# Supplementary material for: Coral-YOLO: An Intelligent Optical Vision Sensing Framework for High-Fidelity Marine Habitat Monitoring and Forecasting
Source: Sensors (Basel). 2025 Nov 29;25(23):7284. doi: 10.3390/s25237284 (PMC12694129; doi:10.3390/s25237284)
Supplement: Supplementary file 1 [file sensors-25-07284-s001.zip › sensors-3980521-supplementary.pdf]

Supplementary Materials

S1. Annotation Guideline for Coral Health States in the CR-Mix Dataset

1. Introduction

This document provides the detailed annotation guidelines used for the creation of the Coral-Reef-Mix (CR-Mix) dataset. Its purpose is to ensure high consistency, accuracy, and reproducibility in the labeling of coral health states. These guidelines were developed through an iterative process involving marine biology graduate students and senior coral reef ecologists. All annotators were required to complete a two-week training program based on this document before commencing formal annotation. The visual criteria established herein serve as the ground truth definition for the four primary health states: (1) Healthy, (2) Sub-healthy, (3) Bleached, and (4) Dead.

2. General Annotation Principles

Object Definition: Each bounding box must enclose a single, continuous coral colony or a visually distinct fragment of a colony. If a large colony exhibits multiple, clearly delineated health states, multiple bounding boxes should be drawn for each distinct region.

Bounding Box Tightness: Bounding boxes should be drawn as tightly as possible around the visible perimeter of the coral colony or the specific health-state region, excluding surrounding substrate (sand, rock) and water.

Occlusion: If a coral is partially occluded by another object (e.g., another coral, a fish), the bounding box should still encompass the estimated full extent of the visible part of the target coral. Annotate only what is clearly visible.

Dominant Health State: If a single colony contains minor, interspersed patches of a different health state (less than 15% of the visible area), the entire colony should be labeled with its dominant health state. For more significant mixtures, multiple boxes are required (see Section 4).

3. Detailed Visual Diagnostic Criteria for Each Health Class

This section outlines the specific visual indicators used to classify each coral health state. Representative visual examples are provided in Figure S1.

3.1 Class 1: Healthy

Color: Displays vibrant and uniform coloration characteristic of the species. Colors can range from brown, green, and yellow to blue and pink, but they should appear saturated and rich. There should be no signs of significant paling or discoloration.

Texture: The coral tissue should appear intact, fleshy, and fully cover the underlying skeleton. Polyps may be visible and extended, giving the surface a textured or "fuzzy" appearance.

Contrast: There is a clear and high contrast between the coral tissue and the surrounding environment.

3.2 Class 2: Sub-healthy (Partial Bleaching/Paling)

Color: The coral appears pale, faded, or "washed-out" compared to its healthy counterpart. The coloration is noticeably less saturated, but the white skeleton is not yet fully exposed. This state can manifest as uniform paling across the colony or as mottled/patchy areas of discoloration.

Tissue: The tissue is still largely present, but may appear thinner or more translucent, allowing the underlying skeleton to become partially visible.

Key Discriminator: The primary distinction from 'Healthy' is the significant loss of color saturation. The primary distinction from 'Bleached' is that pigmented tissue is still clearly visible across most of the surface.

3.3 Class 3: Bleached

Color: The coral appears stark white or bright white, as the transparent coral tissue reveals the white calcium carbonate skeleton underneath.

Tissue: Transparent tissue is still present on the skeleton. This is a critical distinction from 'Dead' coral. The coral structure is intact.

Contrast: The coral exhibits extremely high contrast with the non-white background due to its brightness.

3.4 Class 4: Dead

Color: The skeleton is no longer bright white. It has been colonized by turf algae, sediment, or other microorganisms, giving it a dull, dirty appearance. Colors are typically brown, dark green, or grey.

Texture: The surface appears fuzzy or is covered in a layer of sediment. The fine structures of the coral skeleton may be obscured by algal growth.

Key Discriminator: The primary distinction from 'Bleached' is the absence of living, transparent tissue and the presence of colonizing algae, leading to a dark, non-white coloration.

4. Guidelines for Ambiguous Cases and Adjudication

Healthy vs. Sub-healthy: If an annotator is uncertain, they were instructed to use a "conservative principle": unless there is clear, undeniable loss of color, label it as 'Healthy'. Disagreements on this class required a third, independent annotation and final review by a senior ecologist.

Bleached vs. Dead: The key indicator is the color and texture. A recently dead coral might still appear somewhat white but will quickly be covered by a thin film of algae. If any part of the skeleton shows signs of algal colonization, it should be classified as 'Dead'.

Colonies with Mixed Health States: If a single colony displays large, distinct patches of different health states, separate, non-overlapping bounding boxes should be drawn for each region.

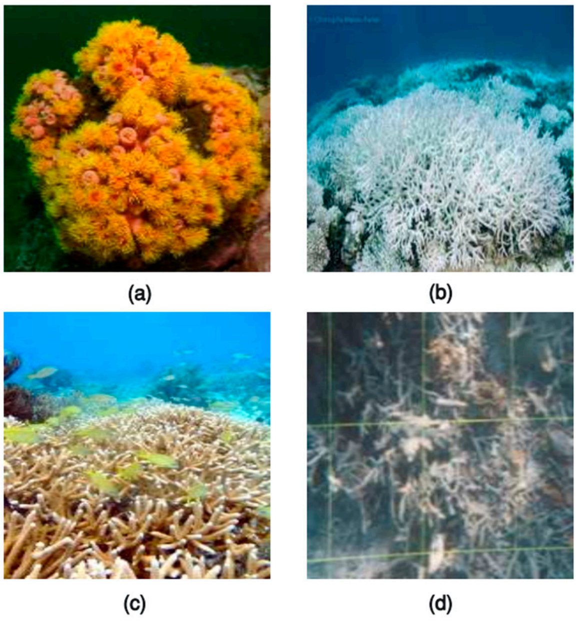

Figure S1. Visual examples illustrating the diagnostic criteria for the four annotated coral health states. (a) A healthy coral with vibrant, saturated color. (b) A sub-healthy coral showing significant paling and loss of color saturation. (c) A bleached coral, appearing stark white as the skeleton shows through the transparent tissue. (d) A dead coral covered in dark turf algae.
